# Development of Sensors-Based Agri-Food Traceability System Remotely Managed by a Software Platform for Optimized Farm Management

**DOI:** 10.3390/s20133632

**Published:** 2020-06-28

**Authors:** Paolo Visconti, Roberto de Fazio, Ramiro Velázquez, Carolina Del-Valle-Soto, Nicola Ivan Giannoccaro

**Affiliations:** 1 Department of Innovation Engineering, University of Salento, 73100 Lecce, Italy; roberto.defazio@unisalento.it (R.d.F.); ivan.giannoccaro@unisalento.it (N.I.G.); 2 Facultad de Ingeniería, Universidad Panamericana, Aguascalientes 20290, Mexico; rvelazquez@up.edu.mx; 3 Facultad de Ingeniería, Universidad Panamericana, Álvaro del Portillo 49, Zapopan Jalisco 45010, Mexico; cvalle@up.edu.mx

**Keywords:** precision agriculture, IoT devices, on-cloud software platform, decision support systems, solar energy harvesting, node power consumption, BLE sensor tag

## Abstract

The huge spreading of Internet of things (IoT)-oriented modern technologies is revolutionizing all fields of human activities, leading several benefits and allowing to strongly optimize classic productive processes. The agriculture field is also affected by these technological advances, resulting in better water and fertilizers’ usage and so huge improvements of both quality and yield of the crops. In this manuscript, the development of an IoT-based smart traceability and farm management system is described, which calibrates the irrigations and fertigation operations as a function of crop typology, growth phase, soil and environment parameters and weather information; a suitable software architecture was developed to support the system decision-making process, also based on data collected on-field by a properly designed solar-powered wireless sensor network (WSN). The WSN nodes were realized by using the ESP8266 NodeMCU module exploiting its microcontroller functionalities and Wi-Fi connectivity. Thanks to a properly sized solar power supply system and an optimized scheduling scheme, a long node autonomy was guaranteed, as experimentally verified by its power consumption measures, thus reducing WSN maintenance. In addition, a literature analysis on the most used wireless technologies for agri-food products’ traceability is reported, together with the design and testing of a Bluetooth low energy (BLE) low-cost sensor tag to be applied into the containers of agri-food products, just collected from the fields or already processed, to monitor the main parameters indicative of any failure or spoiling over time along the supply chain. A mobile application was developed for monitoring the tracking information and storing conditions of the agri-food products. Test results in real-operative scenarios demonstrate the proper operation of the BLE smart tag prototype and tracking system.

## 1. Introduction

Agriculture, by some decades, is undergoing to rapid evolution in methodologies and techniques, for improving the productivity and the environmental sustainability; nowadays, the development of new technologies allows to implement an intensive resources exploitation (e.g., soil, water, fertilizers, etc.), with the aim to produce high-quality fruits and vegetable products in sufficient quantities to satisfy food requirements while maintaining competitive costs. In particular, intensive agriculture allows to satisfy the primary needs of the population in constant growth, but, on the other hand, it has caused serious ecological and environmental damages such as deforestation, desertification, the unregulated use, namely the abuse of pesticides and fertilizers and consequently the ground-water pollution. Therefore, the aim is to achieve sustainable agriculture from the ecological and environmental point of view and obviously also in terms of economic compatibility. A key factor to obtain this result is the correct and optimized use of the various input resources such as fertilizers, water and plant protection products, into the agricultural production processes. Thanks to the development of Internet of things (IoT)-based technologies, nowadays a huge number of low cost and power consumption modules and sensors are available to be employed in the farmlands, in order to detect and collect precise climate and soil data as well as information referred to growth or diseases of the cultivated crops and send by Wi-Fi connectivity all the collected data to a cloud software platform for the optimized management of the farm.

The designed system represents a multi-platform complete solution, by employing a smart fertigation system assisted by an on-field custom wireless sensor network (WSN), for supporting the farmer and manager along the whole supply chain from the field to the product distribution, as well as for providing added value services to the customers. In fact, the developed system helps the dealers and traders to check the transportation and storage conditions of the food products, as well as their provenience, employing a low-power Bluetooth low energy (BLE) smart tag placed inside the food container; therefore, the availability of a single multi–functional software platform allows the user (both the farmer and costumer) to check all events and treatments performed overtime on the products, from the sowing to the consumer table. To exploit the multitude of the offered services from the developed system, an integrated on-cloud software platform allows us to manage the crops production and market of the agri-food products and more.

The benefits of the developed farm optimal management system, integrated with an automated fertigation facility, can cover several cultivation typologies, such as arboreal cultures, horticulture and any type of greenhouses. A low-cost and energetically autonomous fertigation system has been developed to properly dose and mix the determined amount and typology of fertilizer into the water to irrigate. Several fertigation systems, installed in different cultivated lands also far from each other, can refer to the same cloud platform and, thus, to the same agronomic database containing the data derived from the mathematical models for the different considered crops. In fact, the agronomic models allow to determine the optimum cultivation parameters, such as typologies and amount of fertilizers, frequency and time duration of irrigations; all these operations are calibrated based on crop typologies, weather condition and on the physical and chemical quantities, related to soil and air, acquired directly on-field. The decision-making algorithm is also supported by a tree-structured WSN installed on the land, so allowing to obtain updated and real-time data related to the crop conditions. According to the aforementioned functionalities, the proposed system allows us to obtain savings in terms of water resources, fertilizers placed in the ground and energetic consumption, with respect to classic methods, and a complete traceability relative to the cultivated products.

The software platform receives non-stop the WSN data, through the central management unit, from coordinator nodes and the weather forecasts from reliable meteorological websites, respectively and then it utilizes them in the mathematical agronomic models to support the farmers or agronomists in the decision-making processes related to the treatments necessary for the optimal growth or any diseases of cultivated crops. In particular, a low-cost and robust WSN has been realized by using commercial development modules and sensors; besides, through a compact solar harvesting section, and minimizing node power consumption by means of a suitable firmware implementation, long energy autonomy of each sensor node has been obtained.

The decision support system (DSS) implemented in the software platform uses always updated databases on fertilizers and plants’ protection products, including all technical specifications and mode of use that allow the correct selection of the most appropriate product for each specific application or treatment, also concerning the anti-resistance strategies, thus becoming an effective tool for adapting cultural techniques to the climate changes and specific geographical location. In addition, the developed software platform allows the traceability of the agri-food products ensuring the storage of information on the treatments undergone by the products during the cultivation phases, so allowing then the data viewing remotely on the smartphone/tablet of potential consumers interested in purchasing the products. In addition—by using a multi-platform application—the farmer or the agronomist can identify, by reading a specific quick response (QR)-code, a portion of the cultivated land and view or update the related information on cultivated crops in this specific area or finally can acquire data about the performing of a specific treatment on the cultivated crops.

Furthermore, a tracking and tracing system was designed, based on BLE sensor tag applied to the plastic or wooden container, where the fresh or already processed agricultural products are stored. The low-power consumption BLE tag is equipped with several sensors for monitoring the parameters useful to determine the freshness status and the storing conditions of different types of food products (i.e., temperature, humidity, total volatile organic compounds (TVOCs)-level, ammonia (NH_3_) and carbon dioxide (CO_2_)-concentrations). The variety of detected parameters makes the realized device extremely versatile for monitoring different food typologies (vegetables, meat, fish, etc.), as demonstrated in the literature analysis in Section 2.3, a unique feature not found in other competitor systems reported in the literature as well as available on the market. Additionally, the developed BLE smart tag is reusable for a long time on the same container or, if the container is damaged, it can be easily removed and applied on another container, as following detailed in Section 3.3. In addition, the iBeacon technology is exploited to support the tracking system in the monitoring of the agri-food stored products on the whole supply chain (SC), so providing a value-added service to the consumers. Alternating iBeacon phase with low duty-cycle acquisition and transmission phases, the developed BLE device acts like a reprogrammable tag capable to monitor the freshness indexes of the stored food typology. A prototype of the BLE smart tag was tested to verify its correct operation and evaluate the energy autonomy, which turned out to be about 5.6 months. A mobile application was developed to remotely monitor the tracking and storing parameters acquired by the BLE tags placed inside the containers with the agri-food products. Both the BLE tag and tracking system were tested in different real operative scenarios, by using oranges and frozen codfish fillets placed inside plastic and polyethylene containers, demonstrating their correct operation.

Consequently, the developed software platform for the optimized farm management allows implementing an innovative business model inspired by the principles of the products’ traceability, to guarantee consumers on their quality, as well as of the organically grown food and fair trade forms of agri-food products. In fact, the on-cloud software application specifically developed for the development of new business models provides new forms of fair trade of the food products and other implemented services for increasing the potential market of farm agricultural production.

The manuscript is organized as follows: in the sub-paragraph 1.1, a non-exhaustive literature analysis about innovative DSSs applied to agriculture field is reported; these software tools, based on data provided from several sources, help the farm manager to take correct decisions on cultural processes as well as to prevent or minimize effects of pests and diseases or winter frosts. The following paragraph includes an overview of new remotely controlled devices and IoT-based technical solutions developed for crop monitoring and precision farming. After, a literature analysis on the applications of tracking and tracing systems for agri-food products’ identification at the harvest time and subsequent their traceability is reported, so providing a quality certification to the consumers and an economic advantage to the farmer. Furthermore, in this subsection, the most suitable parameters for monitoring the freshness status of rapidly perishable food products (e.g., meat, fish, vegetables, etc.) are identified and discussed. In Section 3, a description of the automated fertigation facility integrated with the WSN located on the farmland is provided with technical details on its functionalities and related to the different mechanical, hydraulic and electrical systems. In addition, the architecture of the developed BLE sensor tag for the traceability system of agri-food products is described, applied to the most commonly used containers, made in plastic, wood or polystyrene, for foods’ transport or storage. In Section 4, the analysis of power consumption related to the sensor node during the different operating phases is reported in order to estimate the lifetime of the used lithium polymer (LiPo) battery recharged by the solar energy harvesting system; in Section 4.1, the operation of the developed tracking system based on BLE sensor tag is also reported. Finally, in Section 5 the architecture of the software platform, both for managing the fertigation system by the agronomist or system manager and for the food products’ tracking is described; besides, the functionalities of the developed mobile application both for the farmer and customer users are discussed.

## 2. Scientific Works and Commercial Devices for Precision Farming and Advanced Tracking Systems

In this section, an overview on recent scientific works related to DSS-based algorithms and IoT solutions applied to precision farming has been reported, together with a state of the art on advanced tracking and tracing systems for monitoring agri-food products.

### 2.1. A Literature Analysis on Sensors-Based Decision Support Systems (DSS) Applied to Precision Farming

In the last decades, also agriculture is benefiting from the technological advances in electronics, computers technology and communication fields [1,2]. In this context, great relevance is ascribable to the DSSs, a wide range of software tools that based on several input data related to soil and environmental parameters, acquired by sensor nodes distributed on the cultivated land, to weather forecasts, to crops’ growth status or pathogens presence, then help the farmer to make decisions relative to farm activities or issues to be addressed [3].

In [4], the authors described a real-time DSS, for minimizing disease effects for the tomatoes cultivated in a greenhouse, structured in three stages: the supervision one detects and diagnoses any failure of sensors involved in culture monitoring, the control stage monitors the climate parameters inside the greenhouse and the management one, based on pests and diseases data acquired on field, optimizes these parameters to limit the spread of pathogens harmful to crops. Furthermore, the DSS integrates a real-time rule-based tool, implemented by G2-Gensym Corporation software and supported by a supervisory control and data acquisition (SCADA) control system; this last has as inputs the actuated variables (windows status, irrigation valves, heating), as outputs the controlled variables (interior temperature and humidity, soil water content, nutrients, etc.), whereas the disturbances are the uncontrolled variables, such as weather conditions, diseases and pest presence. Moreover, a fuzzy-logic controller allows optimizing the system dynamic behavior by modifying the set-point variables of the climate control unit.

**Figure 1:**
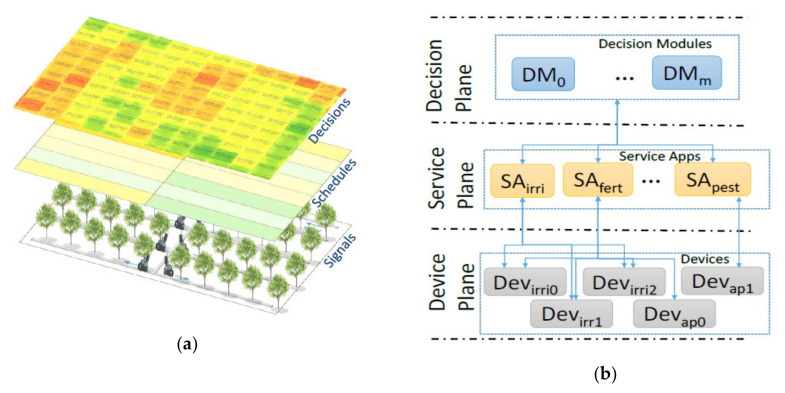
(**a**) Decision support system (DSS) software structure reported in [5] and (**b**) its three-layer architecture with decision, service and device planes, respectively.

An innovative framework for a cloud-based decision support automation system (DSAS) in order to assist the orchards’ cultural practices was presented in [5], able to acquire data from different sources, to make specific decisions and control devices installed on the field. It has a modular software architecture, featured by decision modules arranged in a hierarchical structure, handling the diversity of data by using a data model to specify the format, data types and enabled operations of input data set. Given the heterogeneity of possible cultural operations on the orchard, the cloud-based DSAS has an extendible and adaptive architecture, for adding a new decision module when required. In particular, the software follows the open/close principle, namely, it is open for any extensions, but close for modifications. The software-managed control was structured in three layers: (1) the decision layer defines the actions at a high level, (2) the service plane schedules the actions for each device, (3) the device layer performs the defined schedules (Figure 1).

An IoT-based system for smart agriculture that employs a tree-type decision algorithm was described in [6], using as main elements the IoT devices for acquiring information directly on the field, the gateway and the service platform. The sensor nodes employed the Arduino board for detecting parameters from connected sensors and controlling actuators; acquired data are fused to produce a more consistent and accurate database and, employing Kalman filter, the noise derived from data fusion operation was minimized. A Raspberry Pi board was used as a gateway for connecting WSN nodes to the cloud service. The algorithm employed in the decision-making process is the tree-type one (“*if-then rules*”) based on decision rules and input data both stored in a my-structured query language (MySQL) database.

E. Han et al. proposed a DSS tool, named climate-agriculture-modelling and decision tool (CAMDT) able to combine the seasonal climate forecasts (SCFs) with the crops model, for readily obtaining information related to cultural processes adjustment and water management, thus ensuring improvements of crops yield based on the climatic forecasts during the cultivation period [7]. In particular, the CAMDT was supported by DSSAT (decision support system for agro-technology transfer)-CSM (cropping system model)–Rice model for obtaining tailored information for crops decisions, both in terms of direct outcomes (such as yield and water stress) and translated outcomes (such as the risk of water stress and gross margin).

C.C. Baseca et al. developed a real-time smart decision system to determine the variable rate irrigation parameters and variable rate fertigation rules for optimizing the crops yield [8]; it collects crops data (soil and leaf sensors), weather forecasts, machinery data (pivot irrigation, controller units and actuators, pumps status, etc.), that are sent to a middleware. After a preprocessing phase, these data are processed by means of a decision-making system obtained by combining the learning prediction rules with the Drools rules management system for producing relevant results and thus allowing to improve both farm sustainability and competitiveness.

Torres et al. developed a three-layer data fusion algorithm, called Hydra, for improving the accuracy of employed sensors to determine some critical events more accurately, and thus for making appropriate decisions related to the smart water management [9]; the Hydra algorithm employs, at the lower level, some filtering methods (extreme Studentized method—ESD and weighted outlier–Robust Kalman filter—WRKF) for identifying and removing the outliners, thus enhancing the algorithm reliability.

In [10], the authors presented a robust approach to simulate, by employing cultural simulation models for a specific location and year, the crops yield potential (Yp) and to estimate the yield gap (Yg), difference between Yp and average actual farm yield (Ya). The authors aim to provide guidelines for estimating yield gaps and for simulating the climate change impact and land-use change from local to global spatial scale.

The state of the art on big data applications in smart farming is presented by authors in [11], explaining how new technologies and devices, namely IoT-oriented solutions and cloud computing, are influencing the entire food supply chain, so providing predictive insights in the agricultural processes, driving real-time operational decisions and also redesigning the farm economic processes towards new business models. In [12], the current state of agricultural systems science is shown, focusing on capabilities and limitations of agronomic models that need to be improved; common limitations are scarcity of data for developing, evaluating and applying farming system models and inadequate knowledge systems able to communicate model results to society. New open data initiatives show promising results to tackle the data problem, but there must also be a cultural change among agricultural researchers; the conclusion is that multiple platforms and different models are needed for farming models’ applications for different purposes.

About DSS tools, most of the existing ones are focused on relatively narrow issues, for instance, the application of a specific fungicide, when to apply the next irrigation or how much fertilizer to apply to a particular crop that will be grown on a specific type of soil [12]. One of the problems related to DSS validation is the change of climate conditions at the field scale that can be considered in the DSS only with the implementation of predictive models capable of assessing the response of agricultural systems to the changing of the environmental conditions.

To overcome these limits, the authors in [11] investigated, by means of a DSS agro-technology transfer-cropping system model (DSSAT-CSM), (i) the spatial variability of soybean yields across the farming field with different soils and inputs, (ii) how spatial variability in soil properties translates into measured and predicted yields variability and (iii) how scaling soil properties affects model accuracy in predicting soybean yields for different weather conditions [13].

An example of DSS, named AGRODSS, was recently presented in [14]; implemented as a cloud-based service, it enables various software products for agriculture to facilitate decisions support to users, allowing, as other existing DSSs, to improve business value through savings, avoiding risks, better planning and taking better advantage of available resources. AgroDSS is based on data mining approaches able to extract useful information from large volumes of data by using advanced algorithms to identify trends, distributions and to find patterns that can be hard to see with naked eyes. The newly acquired knowledge can better help understand the data, see how data changes in time, and help to predict the future impact of different actions and farming processes. In [15] the authors described an intelligent logistic tracking and tracing system, able to ensure traced horticultural food security and quality, so determining fair prices. The traceability framework consists of four following phases, namely identification, modeling, processing and traceability data presentation, so helping decision-makers in the issue of food security, obtaining an efficient product distribution and increased customers’ satisfaction. Finally, the developed logistical approach employs fuzzy set theory, artificial neural networks, sensors technology and genetic algorithms.

### 2.2. Advanced IoT-Oriented Technical Solutions for Monitoring and Precision Agriculture

In this paragraph, an overview, regarding the precision farming applications, techniques, and employed Io-T oriented devices, is reported in order to exploit the available resources in the best and most efficient way and also to satisfy the needs of cultivated crops with the ultimate goal of optimizing their quantity and quality.

WSNs development and their growing popularity are due not only to the usefulness for individual users, but also to the role that they can play in supporting the entire community; an interesting example is the forecast service on significant atmospheric events. In this context, the sEmPliCE project (Eledia civil protection expositor), developed by the ELEDIA research center in Trento (Italy), aims to make easily accessible the numerous data related to environmental monitoring acquired by heterogeneous technologies present on Trento’s provincial territory. Through an intuitive software platform accessible by smartphones and tablets, the interested citizens can obtain information updated in real time and consult the data history, receiving alert notifications in case of a forecast of natural disasters.

**Figure 2:**
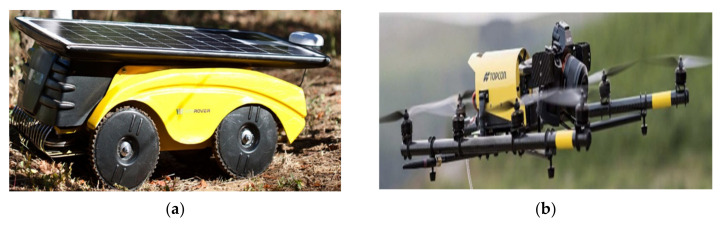
Image of solar-powered Vitirover robot in action on the ground (**a**); Falcon 8 drone employed to monitor, by means of the infrared (IR) sensors, the chlorophyll amount inside the vineyards (**b**).

In the next future, a fundamental role will be represented by artificial vision systems able to manage the actions performed by actuator robots on the cultivated products; concerning this topic, the research has already yielded significant results [16]. Below are shown some examples of robots employed to perform different tasks depending on the specific application and crop typology. In Figure 2a, the so-called Vitirover autonomous mower-robot is used to eliminate herbicides being able to mow the grass up to a few centimeters from the foot of vine plants; energy autonomy is guaranteed by photovoltaic cells placed on its roof (for further detail http://www.vitirover.com/) [17]. The robot works on the basis of the global positioning system (GPS) coordinates, provided by the manufacturer, related to cultivated land limits, being able also to avoid any holes or pits; it is controlled by Please verify. or smartphone app and through it, the farmer can manage the robot by positioning it in the ground or vineyard. In addition, the drones are employed in the agricultural sector for monitoring the crops’ growth or pests’ spread; in Figure 2b, the so-named Falcon 8 drone is shown while it is flying over an agricultural land. The drone, provided with eight propellers, is capable of making video footage and, by using some infrared (IR) sensors, to detect the chlorophyll amount inside the vineyards, a fundamental parameter for agronomists and winemakers.

In the GRAPE (acronym of ground robot for vineyard monitoring and protection) project [18], an autonomous ground robot equipped with a robotic arm was realized to monitor plants health in the vineyards and, by navigating along the rows of vines, to dispense pheromones against pests. Many research works are focused on the possibility to use the emerging digital technologies to improve and aid the agricultural sector management [19]. In addition, the significant potential of big data analysis applied to smart and precision farming is highlighted in [20]; the authors show that the availability of hardware and software techniques and methods for big data analysis, as well as the increasing on-field usage of big data sources, shall encourage more academic research, public sector initiatives and business ventures in the agricultural sector.

From the hardware point of view, the most used technology to detect different interest quantities of agricultural land is that of WSNs that can be employed to carry out several monitoring operations; for instance, they are used for environmental condition monitoring or for providing information about soil nutrients, allowing to predict crop health and product quality over time. A classification related to different study cases to thoroughly explore the existing solutions proposed in the literature, according to their design and implementation parameters is presented in [21]. Specifically, different farming applications that can be implemented by using WSNs are presented in [21] related to irrigation management systems, farming monitoring systems, pest and disease control and prediction, controlled use of fertilizers, vineyards’ production monitoring, groundwater quality monitoring, cattle movement monitoring, gases monitoring into a greenhouse, asset tracking, remote control and diagnosis. In addition, the authors classified the possible architectures in various categories, highlighting the potential agricultural applications suitable for each one.

WSN nodes are based on programmable microcontrollers and designed as modular structures open both from the hardware and software point of view [22,23]. The sensors used for detecting different environmental and soil parameters have to be carefully chosen; concerning soil water content measure, numerous techniques are currently in use and the suitability of each one depends on cost, accuracy, durability and response time [24]. A widely used measurement technique is based on the dependence of the dielectric constant with the soil water content that significantly affects the propagation of the electromagnetic waves inside the medium; in fact, the dielectric constant of dry soil is much less than a wet one. Two approaches are commonly used to measure the dielectric constant of soil, namely the time domain approach and frequency domain one; advantages of the first are the low cost, need of low-frequency circuits, no employment hazardous radiations and a fast response time. Instead, the frequency domain approach has higher sensitivity to insulation and is more sensitive to air gaps. A low-cost solar-powered monitoring station equipped with an analysis probe based on the frequency domain method was developed in [25] for measuring temperature and humidity of both air and high silica content clay soil at different depths.

A proper WSN design represents a fundamental point for monitoring different parameters in an agricultural land; a significant issue that has to be taken into account regards the power consumption of each sensor node, for ensuring long node autonomy. In this regard, energy harvesting techniques represent a real opportunity, thus obtaining a technical solution nearly with no maintenance [26,27,28,29].

Air pollution monitoring is another field where WSNs are employed; the meteorological conditions, such as wind speed and atmospheric turbulence, affect air quality as reported in [30] where a WSN was designed, with an effective energy-saving system for each sensor node, for air and environment pollution detection. In this context, in [31] the authors propose a low-cost WSN for air monitoring applications; the sensor node is based on commercial gas sensors and a low power ZigBee (zonal intercommunication global-standard bee) module, which shares the acquired information with the cloud by means of a gateway. The experimental results demonstrate the proper operation of the node, having obtained a 93.03% discrimination rate and R^2^ coefficient values, in the gas quantitative detection, around 0.99. An overview of WSN applications is presented in [32] focusing on five application areas, namely healthcare, agriculture, environmental, industry and military. Moreover, the technical features of both hardware and software sections for WSN design are given in detail depending on the application [32]. A WSN-based smart system was developed in [33] for monitoring the availability level and rapid changes of water content in the soil, with the aim of guaranteeing early flood prediction, water savings in the farmland irrigation as well as waste reduction and optimal use of water resources where its availability is poor [34,35]. All aforementioned WSN solutions can be applied in the precision agriculture field, namely a management strategy that employs information technology to improve crops’ quality and production [35,36]. Wireless sensor technologies and Information Technology (IT)-based management tools can lead to a highly effective green agriculture, including, just as an example, the proper supply of adequate nutrients to crops and appropriate use of pesticides for the optimized control of weeds, pests and diseases.

The recent WSN applications in precision agriculture are analyzed in [37], where the various wireless communication protocols are classified and compared; besides, this work reports a study on the taxonomy of energy-efficient and harvesting techniques for WSNs used in agricultural monitoring systems and a comparison between previous research works on agriculture-field WSNs. In addition, the deep learning, a recent modern technique for image elaboration and data processing, was introduced in agriculture domain; specifically, in [38] a survey on 40 research works employing deep learning techniques in various agricultural and food production areas was carried out; in this context, the authors analyzed particular agricultural issues, namely the used frameworks and models, the sources, nature and preprocessing of the collected data and the overall performance achieved according to the metrics employed in each under-analysis research work.

The optimization of energy consumption is crucial in WSN applications for agriculture since usually the nodes are distributed in remote areas where the maintenance for changing or recharging the batteries is difficult. Del Valle-Soto et al. developed a WSN energy model to estimate the energy consumption of each node during the different operations (data acquisition and transmission), when the routing protocol is running [39]. The model validation is performed by employing TI CC2530-based boards, which run a multiparent hierarchical (MPH) routing protocol. The experimental results indicate that the proposed protocol shows a lower energy consumption respect to ad-hoc on-demand distance vector (AODV), ZigBee tree routing (ZTR) and dynamic source routing (DSR), whereas the MPH protocol has a slightly greater energy consumption compared to the energy-aware protocols such as power-efficient gathering in sensor information systems (PEGASIS) and low energy adaptive clustering hierarchy (LEACH) (2% and 3%, respectively).

A crops’ monitoring system based on wireless sensor network is reported in [40]. The authors present their developed system composed of seven parts: the data processing unit (model ATmega128, manufactured by Microchip Technology, Chandler, AZ, USA) which processes all the information and manipulates the peripherals, the CC1101 Radio Frequency (RF) module (manufactured by Texas Instrument, Dallas, TX, USA) for transmitting data collected by sensors related to environmental and soil parameters, a control unit to regulate the activation/deactivation of each employed sensor node (for obtaining energy saving), a data storage unit composed of AT45DB041D serial-interface flash memory (manufactured by Adesto Technologies, Santa Clara, CA, USA), a power supply unit (DC source or solar battery), an analog interface used to adjust voltage values provided by the employed sensors as required by the analog to digital converter (ADC) and finally a digital interface for digital sensors. In their system, the authors employed also an image sensor node made up of a system-on-a-chip (SoC) THLK2405 images processing module and a CMOS model OV7640 images array sensor (manufactured by OmniVision, Santa Clara, CA, USA) capable of operating at up to 30 frames per second (fps) [20].

In the proposed system, the WSN is fully integrated with an innovative fertigation unit and a farm management software platform implementing a DSS-algorithm supported by agronomic database; also, the software platform allows the user, by means of a mobile application, to control all the system functionalities and to view the stored data concerning the carried out treatments and irrigations, as described in Section 5.

### 2.3. Potentialities of Tracking and Tracing Software Systems and Analysis on the Most Suitable Parameters for Monitoring the Freshness of Rapidly Perishable Agricultural and Food Products

As known the supply chain (SC) of agriculture products involves several processing and management steps before the products could reach the final users; each step has much data associated with it, often unknown to the end customer. However, these data could be crucial both for companies, in order to create logistic strategies and marketing techniques, but also for the end costumers to ensure food security and quality. For these reasons, exploiting the IoT advances, different smart tracing and tracking systems have been developed. The agri-food industry is one of the most promising candidates which could benefit from all the features introduced by these technologies. Specifically, intelligent packaging (IP), radio frequency identification (RFID), WSNs, analytics and cloud platforms have been pervasively applied to tracing and tracking systems, allowing to improve the tracing capabilities along the whole SC, while ensuring the availability and accuracy of products data. Furthermore, the system scalability is allowed by employing analytical methods and big data techniques, making the work of operators easier and more efficient [41].

Relatively to the food chain, almost a third of produced food for human consumption worldwide—approximately 1.3 billion tons every year—is wasted. In industrialized countries, more than 40% of losses happen at the retail and consumer levels. Food is sometimes thrown away ‘just in case’ when it is still fine to be consumed. Different solutions have been developed to monitor the quality of the packaged food and stored before being consumed, ranging from color changing labels as a function of food container temperature to RFID tags that link up with smartphones to give detailed information on the food status and sensors that indicate how ripe fruit is [42]. Poor chill chain management within the food supply chain and some uncertainty about how long ago food packs were opened after purchase are crucial aspects that can determine the failure of stored food products; different companies have developed smart tags that help retailers and the restaurant industry for addressing this issue.

**Figure 3:**
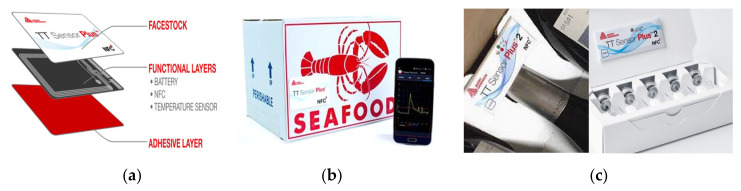
(**a**) Structure of the TT Sensor Plus^TM^ smart label (**b**) to monitor the temperature of food products made available through NFC by using the mobile app; (**c**) TT Sensor Plus^TM^ 2 label applied to a bottle of wine (left) or a pack of drugs [43,44].

In the field of tracking and tracing of food products, an interesting commercial application is the TT Sensor Plus^TM^, which combines a low-power temperature sensor and data logging functionality in an adhesive label [43] (Figure 3a); it can be applied to the container for monitoring the product temperature, making the latter available to the user through near-field communication (NFC) by using a mobile application (Figure 3b). In addition, the evolution of this product, called TT Sensor Plus^TM^ 2, is equipped with two light emitting diodes (LEDs) for indicating the label status (active/inactive) or alert conditions [44]. The device is fed by a 3 V lithium battery, so guaranteeing a long autonomy (more than 3 years after the activation). Such devices are suitable for several applications, as monitoring of pharmaceutical and medical products (Figure 3c), food and beverage, chemicals and polymer-based goods and in general other products sensitive to the storing temperature.

In [45], the authors proposed a multilayer and technological framework to support the company activities in the development of a traceability system based on Industry 4.0 principles. At first, the proposed framework, given the adopted business model, identifies and models the process influencing the product. In addition, the identification of each productive process step and entity which stores the products information along the SC is required. These operations are enabled by the IoT technologies [46,47]. Specifically, the information related to the production environment, to the product transformation or to the transport conditions are acquired and monitored by sensors placed along the whole SC. Moreover, an IT application is employed for collecting these data and making them accessible by the company as well as the end-users, to guarantee the benefits above described.

Qi et al. illustrated a WSN-based integrated cold chain shelf life decision support (C^2^SLDS) system, designed for perishable food products cold-chain management [48]. The developed architecture relies on a WSN for acquiring and transmitting, in a distributed manner, the products’ parameters, while a time–temperature indicator (TTI) monitors and records the time–temperature history of the products along the whole SC. The system consists of a 3-layer architecture, named WTTI (WSN-based TTI), AGS (advanced RISC machine (ARM)-based gateway system) and LDSS (least shelf life first out (LSFO) decision support system), respectively. The WTTI is a reduced-function device, able to acquire temperature and wirelessly transmit shelf life information to a ZigBee coordinator. The AGS consists of a Zigbee coordinator node and an ARM-based smart device that gathers the temperature data from the coordinator node. The LDSS decisions are driven by the application server, the data warehouse server, the model base, the knowledge base, the routers and the firewalls. Based on this information, a software application calculates the shelf life of the products by means of the TTI, once known the shelf life prediction model and the parameters in the knowledge base.

The afore reported works propose devices able to monitor, each, only one physical or chemical quantity, so indicating the freshness of the specific stored product; instead, the designed BLE smart tag is able to simultaneously detect several physical and chemical quantities, so covering a wide range of applications, for monitoring the storage conditions of different food typologies.

In [49], the author proposed a traceability system applicable to the agri-food products supply chain. This system employs RFID technology for enabling the data acquisition, the traceability during the different productive steps (production, warehousing, distribution) as well as the sale links of agri-food products. In addition, the system employs several blockchain technologies to ensure the authenticity and reliability of the shared information. Another approach to traceability of vegetables based on RFID technology was described in [50]; specifically, a unified modeling language (UML) is defined to support the product traceability and, thus, creating a method for modeling the products information (harvesting place, performed treatments, places of storage, etc.) and quality. Such traceability code was formatted according to the European article number (EAN) and uniform code council (UCC) standards. This UML modeling is used to perform real time tracking of the product, thus allowing it to easily improve its quality as well as reducing the waste.

**Figure 4:**
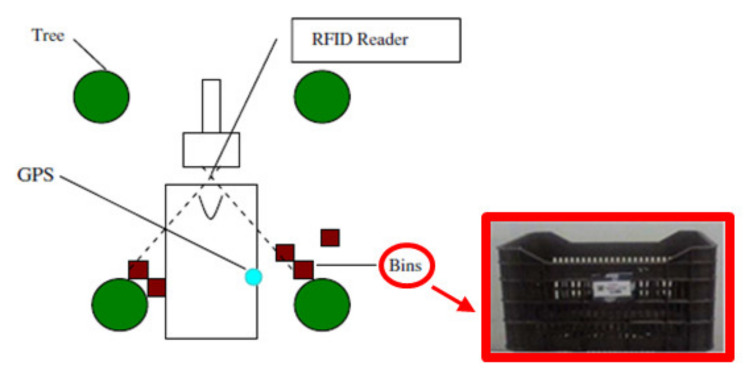
Representation of bins collection by the tractor equipped with a radio frequency identification (RFID) reader and differential GPS (DGPS) receiver.

Ampatzidis et al. proposed two solutions to bind the bins employed to harvest the fruits with the corresponding pairs of trees, thus establishing the harvesting yield of an orchard [51]. The first method relies on passive RFID tags, operating in the ultra-high frequency (UHF) band (866 MHz for Europe), placed on the bins (Figure 4) and a long-range RFID radar-reader (manufactured by Trolley Scan Company^TM^, Johannesburg, South Africa) and a differential GPS (DGPS) receiver (model AgGPS^®^ 106, manufactured by Trible Geospatial, Vimercate, Italy), located at the back of the collecting tractor, 1.50 m above the ground; in the second one, only the RFID tags are used, which are placed onto each pair of trees as well as bins.

In addition to the implementation of a traceability system, the low-cost BLE tag, designed and tested in this research work as reported in the following sections, allows the detection of several freshness indexes for monitoring storage and transport conditions, making them available to the user by a proper mobile application.

In [52] two examples of the integration between RFID technology and WSNs to trace any step of the SC of the wine production from the vineyard to the final consumer are proposed. This allowed demonstrating how the constantly growing need to know more about the food we eat can be satisfied, successfully monitoring and storing for traceability purposes, all the valuable information of the whole SC. In [53] a similar approach is generalized to demonstrate its applicability to different types of food industries. It has been shown how the integration between WSNs and passive RFID technology could enable traceability improvements for the final customer as well as for the company, maintaining a constant information line along the production and distribution chain, taking care of some critical parameters like events in development, production parameters (for example temperature, humidity, etc.), limited storage life, storage and transport conditions,, etc. All these improvements guarantee a medium-term return of investment for the company as well as a value-adding for the customer.

On the other side, an approach to enhance RFID technology, with the aim to use it for traceability and parameter monitoring, is to integrate sensing capability directly on the tag. The design and actual use of chemical or biologic sensors as well as indicator labels in smart and active packaging, in order to monitor the degree of freshness or correct conservation of food, is a growing field of study, thus obtaining at the same time a real-time food-status monitoring, a reduction of food waste, an extension of shelf life and improvement of overall food quality [54]. For example, in [55] the possibility to exploit some new chemical interactive materials (CIM) to realize battery-less RFID sensor tags, capable to detect different gas species or humidity, is explored. Specifically, considering applications to prevent food-wasting, the use of the doped poly(styrene-sulfonate) (PSS) as CIM to detect ethanol should be evaluated because it is one of the indicators of food perishing. Other chemical and physical parameters can be monitored, and consequently different types of sensors can be used, to determine if food is going to decay. More in detail, in [56] a review of the most widespread chemical and biologic sensors for detecting food quality is presented. Different sensors work properly by monitoring different indicators for specific types of food, in order to control freshness markers or detect the presence of allergens, pathogens, adulterants and toxicants. For example, in fish products, one of the main freshness indicators is the hypoxanthine, which is produced by the metabolic degradation of adenosine triphosphate (ATP), whereas an indicator of the stored grain spoilage is the emission of CO_2_ as result of insects’ infestations. The stored cereals are also often threatened by mildews and fungi, which can be discovered by specific biosensors for mycotoxin detection; instead, concerning fruits and vegetables, volatile organic compounds (VOC) accumulate when they are closed into containers or packages.

The temperature and humidity can also be useful for monitoring food spoilage, not to mention that temperature is a key–value when it is needed to maintain the cold chain. In this regard, some of the authors have already presented in [57] a UHF RFID sensor tag suitable for cold chain applications. Specifically, the device is a semi-passive tag that includes an antenna matched with the EM4325 RFID chip impedance, a micro-controller unit (MCU), LEDs and general purpose input output (GPIO) ports, to provide both tracking and temperature monitoring of fresh and frozen fish. The device was thought of like a reusable board attached to the storage box. The tracked data are collected by the system each time the electronic product code (EPC) is read, but if a temperature threshold value is overcome for a specific time interval, a LED on the board is also lighted up providing a visual alert for the human operator.

Another example of RFID technology applied to food tracking and monitoring industries is proposed in [58], where a printed NFC sensor tag was realized, capable to detect the trimethylamine (TMA), another indicator of the fish and meat spoilage. The authors performed a practical test by successfully measuring the sensor response to the aging of a codfish filet, monitoring the changes in the sensor response from 2 days before to 3 days after the expiring date. A similar proof of concept has been provided in [59], where a smart RFID tag for monitoring the meat decay is described. As the first step, the authors combined different sensors to map the ammonia values when temperature and humidity conditions of the pork meat sample varied. This procedure allowed to discover 4 levels of ammonia concentration for accurately indicating the freshness of under-test food; then the prototype board, integrating the UHF RFID tag, MCU and the ammonia sensor has been developed.

Chemical and physical indicators are not the only ones that can give information about the good conservation of food [60]. Food color is also a good quality marker, especially for food industries where the product appearance is as important as its taste. An interesting example of sensor tag integrating a spectral fingerprint sensor capable to detect not only red–green–blue (RGB) frequencies, but also infrared and ultra violet (UV) light is shown in [61]. The authors realized an FR4 printed circuit board (PCB) with all components of the sensor tag such as the UHF dipole antenna, RFID chip, RGB sensor, IR sensor, UV sensor and the MCU. Its possible application is in the food industry for the detection of the different food colors, indicating variations in food quality, for example, the darker part of a fruit that is going to perish.

In [62], the authors presented different tools and devices (namely sensors, indicators, barcodes and RFID devices) used in the foodstuffs’ intelligent packaging and explained their role and features for maintaining the quality of different food items in terms of both low microbial growth and gas concentration and for providing convenience and easiness to users. The review study also discussed other packaging technical solutions in the supply chain management of food products to control theft and counterfeiting conducts and to improve the company position on the market in terms of branding and marketing. As known, sensor tags require an anti-collision protocol to avoid collisions with other tags present in the same area, as happens, for instance, in a warehouse containing numerous tagged products; for this reason, in [63] the authors propose a new anti-collision protocol based on fuzzy frame slotted aloha (FFSA) architecture and a novel dynamic frame slotted aloha (DFSA) policy. The reported results obtained by performed simulations demonstrate a reduction of the reading time in conditions of a high density of sensor tags.

Most of the devices above described are disposable, ending their life once the tagged product is delivered to the food shop or wholesaler; instead, the realized BLE tag is reusable for a large number of times by picking up the empty containers to be reused by the agricultural producer, as described in detail in Section 3.3, thus allowing to amortize the tag cost over a long time.

## 3. Materials and Methods

### 3.1. Design, Functionalities and Operating Modes of Automated Fertigation Facility Integrated with the WSN

In this paragraph, the functionalities and operating modes of designed solar-powered farm management facility are described, useful for farmers to monitor and control their productions, to command or simply control the cultivation processes performed on crops, such as fertigation cycles or disinfestation by pathogens and able to allow the farmer or consumer to trace the cultivated products in all the phases following the harvest. The fertigation section represents only a component of the whole multi–functional system, fully integrated with the farm management software platform that implements a DSS algorithm for controlling all growth phases of the cultivated products from planting to harvest. The great benefit of the proposed farm facility is also the integration with a custom traceability system, for monitoring the storing and transportation conditions of the food products starting from their collection.

**Figure 5:**
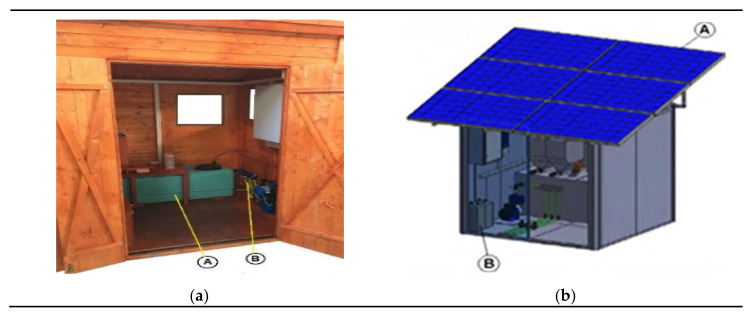
(**a**) Inside the realized wooden cottage with mechanical and electronic sections of the automated fertigation facility, highlighting the (A) mixing tank and (B) solenoid valves for selecting the land sectors to be irrigated; (**b**) 3D image of the solar-powered wooden cottage with the fertigation system inside, indicated by arrows the (A) solar panels positioned on the roof and (B) batteries for solar energy storage.

**Figure 6:**
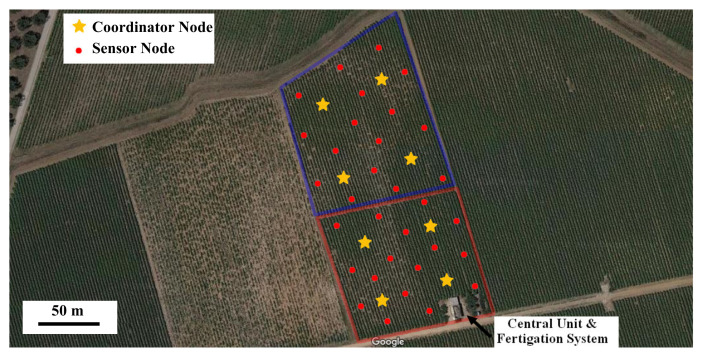
Distribution of the WSN sensor and coordinator nodes on the land cultivated with vineyards in the south of Italy (specifically north of Lecce city), divided into two sectors with area of one hectare each.

In Figure 5a, the solar-powered wooden cottage with the automated fertigation system inside is shown; the hydraulic mechanical section includes up to 5 containers with inside different powdered fertilizers, each provided of dosing mechanism, based on a properly sized cochlea, for precisely calibrating the amount of fertilizer to be added to the aqueous solution (Figure 5b). In this way, the desired amount of fertilizer is added to the water in the tank; after, a mechanical mixer actuated by an electric motor is used for mixing the fertilizing solution. Following, this last is pumped by an electric pump towards the different land sections that require the fertigation process. The composition of fertilizing solution is determined based on agronomic model specific for the considered crop; this model has as inputs the weather forecasts and especially soil and environmental data detected directly on-field by means of the designed WSN. In fact, each WSN node constantly monitors soil and crops’ parameters related to a specific section of the cultivated land and wirelessly transmits the detected data, through its coordinator node, towards the cloud software platform (as shown in the real implementation of Figure 6). This last elaborates received data for determining the correct nutritional and water requirements of the plants belonging to the considered section, as a function of crop typology and growth phase according to the dynamic agronomic database included in the cloud platform. The mathematical forecasting models, included into the developed software system, can forecast, depending on the set treatments and crops growth status, some advance in time or delay relative to the crops’ harvests; the user farmer can activate this functionality according to the purchase orders, received on the farm web portal by consumers and therefore based on the scheduling of deliveries of the agri-food products to be made.

**Figure 7:**
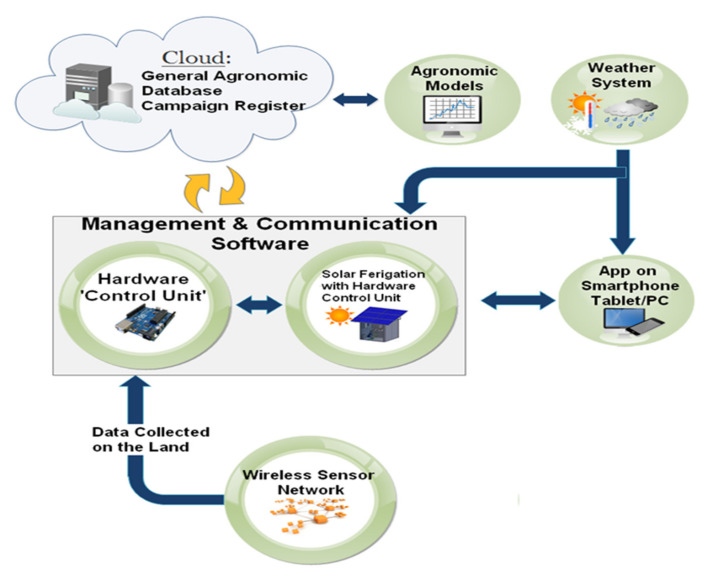
Functional scheme of developed fertigation system with the different hardware and software sections and related data-flow between them; the collected data on the land are processed by the control unit and then shared with the remote general agronomic database in order to be combined with the agronomic and sustainability models as well as weather conditions or forecast.

As shown in Figure 7, the main section of the developed remotely controlled farming facility was the “hardware control unit” which includes both mechanical and hydraulic sections controlled by the electronic unit, thereby allowing to obtain optimized fertigation cycles in each section of the cultivated land, according to the crops’ needs defined by sensors data of the specific WSN cluster on the basis of agronomic models and weather forecasts. The “hardware control unit” was fully powered by a photovoltaic plant consisting of three poly-crystalline 200-W solar panels placed on the wooden cottage roof, a charge regulator (model Giaride 20A solar charge controller, manufactured by Giaride, Inc., Hochdorf, Switzerland), the DC/AC converter (model EDECOA 3000 W, manufactured by EDECOA, Inc., Shenzhen, China) and accumulation battery (model LAGM-160, manufactured by Leoch, Inc., Shenzhen, China) for storing the produced energy; in addition, a control and protection panel was properly sized for protecting the implant against voltage overloads and short-circuits. The designed “control unit” collects information from weather service and on-field WSN related to the environmental and soil parameters and compares them with proper thresholds derived from the agronomic models, thereby defining the fertilizing solution composition for each cultivated crop typology. Hence, this unit properly drives the actuators, belonging to mechanical and hydraulic sections, for obtaining the estimated fertilizing solution and distributing it to desired sections. Another section of the developed farming facility is the cloud software platform containing the general agronomic database, related to the cultivated crops and derived from the agronomic models that determines the threshold values used by the fertigator during its operation.

The fertigation system operations are determined, also, depending on the data directly gathered on-field by means of the developed WSN; this last is constituted by several sensor nodes, with different functions, located in different points of the cultivated field or greenhouse, for detecting environmental and soil parameters as well as data on crops growth. Each sensor node is fed through a photovoltaic system constituted by a small amorphous silicon solar panel, the LTC3105 DC–DC voltage regulator (manufactured by Linear Technology, Norwood, MA, USA) to recharge super-capacitor or lithium battery properly dimensioned for getting long energy autonomy.

The WSN is arranged according to a tree structure, including nodes equipped with sensors for acquiring soil and environmental data and coordinator nodes (or middleware nodes) that collect the data from sensor nodes and transmit them towards the central unit of the fertigation system.

Referring to the agricultural land shown in Figure 6 employed for the cultivation of a vineyard with a total area of just over two hectares, the realized WSN was tested by placing a sensor node every 500 m^2^ (i.e., 20 nodes distributed for each one-hectare sector, as depicted in Figure 6), all connected, through the coordinator nodes, to a single control unit, that manages the fertigation processes. In this way, two neighboring nodes were spaced up to a maximum value of 25–30 m; such distance was chosen on the basis of some communication tests performed also on similar cultivated fields with plants or crops having a maximum height of 70–80 cm and thin stem, in order to guarantee a low packet loss percentage (<2%). However, the number of sensor nodes has to be chosen according to farmer needs, (e.g., if more crop typologies are placed inside the same sector), taking into account that the NodeMCU board ensures a communication range up to 70–80 m in open-field condition or up to 45–50 m in presence of small plants (as above specified). On the other hand, the realized WSN was tested inside an orchard (in particular cherry trees with 3 m average height), at the beginning keeping the same distance between the nodes (30 m), so obtaining a consequent increase of the packet loss percentage (≈20%) mainly due to the path loss caused by the obstacles; in this scenario, the distance between the nodes has to be reduced up to 20 m, for reducing the packets loss percentage to a reasonable value (<2%).

As mentioned above, in Figure 6 the distribution of sensor and coordinator nodes in a vineyard in the south of Italy (specifically north of the Lecce city) is shown, to detect soil and air parameters and carry out the communication tests. The land is divided into two one-hectare sectors with twenty nodes for each one. Five sensor nodes (of the overall 20 nodes for each hectare) are configured to act also as coordinator nodes (or middleware nodes), gathering the data packets provided by the nearby sensor nodes and periodically retransmitting the data, arranged into a multidimensional matrix, to another coordinator node closer to the central unit placed on the underside of the land, until reaching the latter as following detailed.

However, to optimize the WSN topology and reduce its cost, the number of coordinator nodes can be reduced; consequently, longer distance between them and the central unit has to be covered. In this case, a coordinator node equipped with an external antenna is required; thus, as an alternative to the NodeMCU board, a WEMOS D1 mini board (manufactured by Shenzhen RobotLinking Technology Co., Shenzhen, China) equipped with a +5 dBi antenna was tested in these conditions allowing a higher communication range. Regarding the WSN implementation as shown in Figure 6 for its on-field testing, since the average distance between the different nodes is less than 45–50 m (namely about 30 m) without the presence of large obstacles, both sensor and coordinator nodes were realized by employing the ESP8266 NodeMCU board.

In addition, the central unit implements a mechanism for detecting malfunctions or connection problems of each sensor node; in fact, if for five consecutive observation intervals (namely after two and half hours), a given sensor node doesn’t transmit the sensors data, an alert message is sent to the cloud platform, which warns the operator to check the node conditions.

All the functionalities of the developed IoT-oriented farm management facility are governed by a suitable software platform, enabling the farmer or agronomist automatic management of the fertigation of controlled crops, the remote actuation and monitoring of the specific agricultural treatments, but also the purchase of agri-food products by the consumers, as discussed in Section 5.

### 3.2. Sensor Node Architecture and Related Operating Modalities

Communication networks have evolved towards the IoT sector; currently, the most widely used technologies are SigFox, LoRa, BLE, Zigbee and Wi-Fi. Depending on the application, one must consider their performance metrics to choose one technology over another. For example, if technology maturity is the metric, Lora can be considered in a low state, Zigbee in a medium state and Wi-Fi in a very high state. Instead, if the availability is the metric, then Lora and Zigbee exhibit a low level while Wi-Fi a high level [64]. Specifically, in WSNs applications in precision agriculture, Wi-Fi technology is an excellent option, even more so if the Wi-Fi connection is established by employing the low-cost ESP8266 module that has an integrated chip with Wi-Fi connection and supports the transmission control protocol (TCP)/IP protocol. The main objective is to provide any ESP8266-based node with access to the Wi-Fi network and act as an acquisition, processing and Wi-Fi communication board. In addition, in order to offset the energy expenditure of Wi-Fi technology, this module has a low-consumption architecture that allows itself to work in 3 different modes: active mode, sleep mode and deep sleep mode, as detailed and implemented in the following sections.

**Figure 8:**
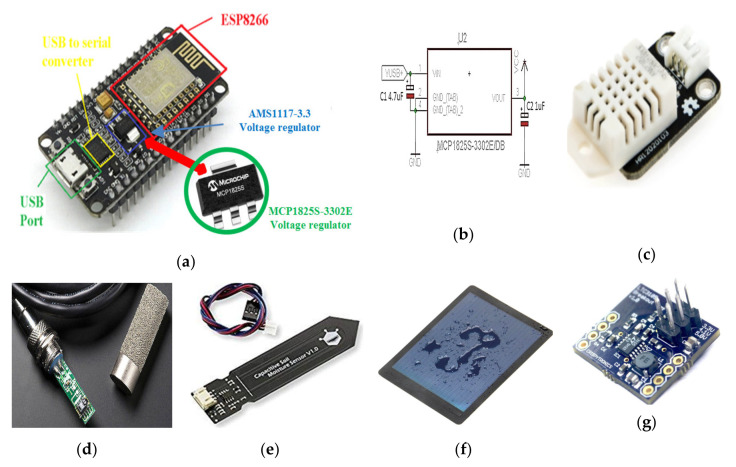
View of ESP8266 NodeMCU module with highlighted the main components included (**a**) AMS1117-3.3 voltage regulator replaced with the MCP1825S-3302E one; (**b**) schematic of MCP1825S-3302E voltage regulator used for reducing node power consumption; (**c**) DHT22 temperature and humidity digital sensor; (**d**) SEN0193 capacitive moisture sensor (DFRobot); (**e**) SHT11-based soil temperature and moisture digital probe (Sensirion); (**f**) 1.5-W amorphous silicon solar panel; (**g**) LTC3105 energy harvester breakout (by Crispytronics).

The designed WSN is constituted by sensor nodes, equipped with several sensors for acquiring soil and environmental parameters, wirelessly connected to the corresponding coordinator node that collects data from cluster nodes and sends them to the control unit of the farm management system, to be immediately shared on the cloud software platform. Both node typologies are based on the ESP8266 NodeMCU electronic board that includes ESP8266 Wi-Fi chip; several commercial sensors were connected to acquire soil and environmental parameters. The NodeMCU was chosen since it combines, into a single device, microcontroller functionalities for data acquisition and processing, as well as a Wi-Fi transceiver for the communication between the WSN nodes; also, it includes a power management unit (PMU) supporting different sleep modalities, which have allowed us to minimize the node power consumption. Specifically, a DHT22 digital sensor (manufactured by Aosong Electronics, Guangzhou, China, Figure 8c) was used to detect environmental temperature and humidity, a SHT11-based digital probe (manufactured by Sensirion, Stäfa, Switzerland, Figure 8d) equipped with a transpiring metallic casing to acquire the soil temperature and moisture and finally the SEN0193 capacitive sensor (manufactured by DFRobot, Inc., Shanghai, China, Figure 8e) for soil water content; also, the SEN0193 moisture sensor is covered with an anti-corrosive layer, allowing long periods in contact with the ground without any damage. The transfer function of the SEN0193 analog sensor was experimentally determined and then implemented in the microcontroller firmware to obtain, from the analog sensor response, the soil water content.

The sensor node autonomy is guaranteed by a storage device (a 100-mAh lithium battery or 90-F super-capacitor) fed by a small flexible photovoltaic panel, able to ensure up to 9 days of energy autonomy (without any availability of solar energy, surely an extreme unrealistic eventuality). Particularly, the used amorphous-silicon waterproof solar panel (manufactured by Dongguan City Xinliangguang New Energy Technology Co., Guangdong, China, Figure 8f) is featured by a 1.5-W maximum deliverable power, 2.0-V open-circuit voltage, 1200-mA short-circuit current and about 10% conversion efficiency. A DC-DC regulation circuit (breakout board designed by Crispytronics, San Diego, CA, USA) based on the LTC3105 step-up converter (manufactured by Linear Technology, Norwood, MA, USA) was used to interface the solar panel with the storage device (Figure 8g); this last has represented either by a 90-F super-capacitor (model Vishay HVC 90 F/5.6 V, manufactured by Malvern, PA, USA) or a rechargeable 100-mAh LiPo battery (model Go Wireless^®^ 100-mAh LFT401430, Beaverton, OR, USA).

In order to reduce the sensor node power consumption, some structural modifications were performed on the ESP8266 NodeMCU board (manufactured by Espressif Systems, Shanghai, China); particularly, the main one concerns the replacement of the proprietary ESP8266 NodeMCU voltage regulator (model AMS1117-3.3, manufactured by Advanced Monolithic Systems, Livermore, CA, USA) with the MCP1825S-3302E model (manufactured by Microchip Technology, Chandler, AZ, USA, see Figure 8a) featured by a lower quiescent current (typical value I_Q_ = 120 µA) and dropout voltage (typical value ΔV = 210 mV for I_LOAD_ = 500 mA), respect to the same quantities of the initial one (I_Q_ = 5mA and ΔV = 1.1 V for I_LOAD_ = 800 mA). Thereby a significant saving in sensor node power consumption was obtained during the deep sleep mode in which the voltage regulator current consumption is the main contribution.

**Figure 9:**
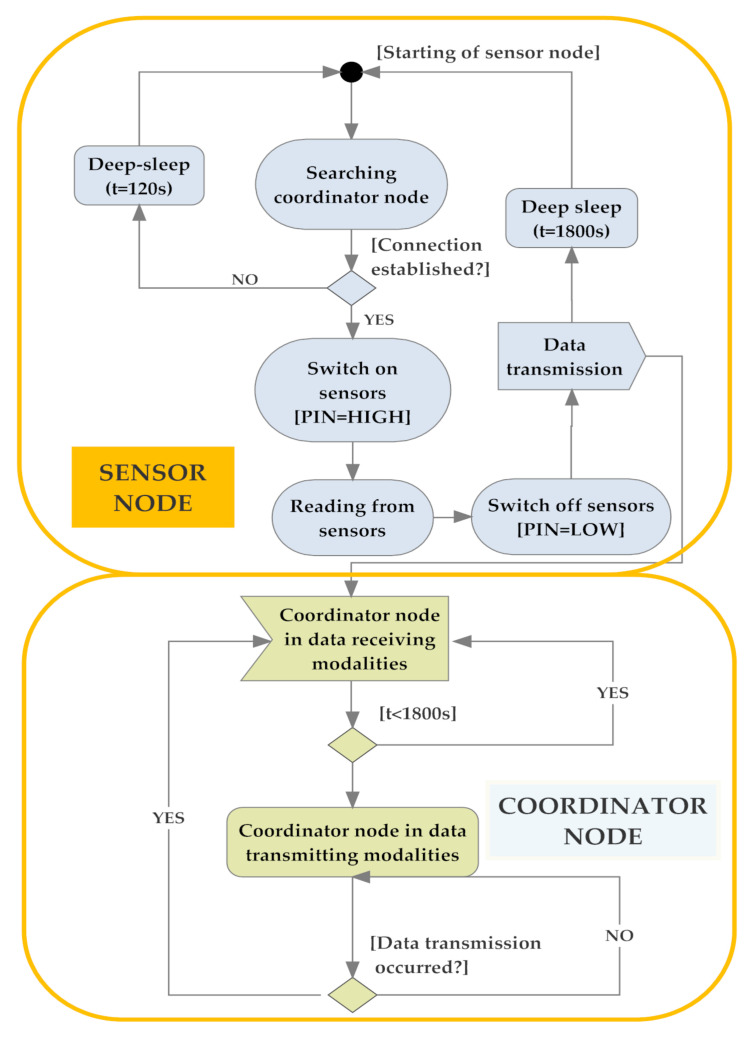
Flowchart related to operations performed by a generic sensor node and the corresponding coordinator node.

Moreover, because the most energetically expensive phase is the transmission one between sensor nodes, to extend node autonomy, WSN operation was properly optimized according to the flowchart of Figure 9, that describes the operating modalities of both sensor and coordinator nodes. Each sensor node acquires the soil and environmental parameters from sensors and sends them to the relative coordinator node; at the transmission end, the sensor node enters in deep sleep mode for 30 min (time interval settable by user) for reducing its power consumption. If the connection with the coordinator node fails, the sensor node retries this operation every 1 min until the connection is obtained. During the connection phase, sensor nodes belonging to a given sector send acquired data to the corresponding coordinator node; every 30 min this last arranges collected data into a single packet and transmits it to the electronic control unit to be sent, via an Internet connection, to the cloud software platform, and then returns in the receiving mode.

The proposed farming facility is constituted by low-cost solutions, but reliable at the same time, in all its sections compared to other competitor systems. The cost of the fertigator and WSN nodes is a function of the land size; for instance, supposing a two-hectare field, the cost of the fertigator is about 2900–3200 €, including mechanical, electronic and hydraulic sections. For the WSN, the cost of each sensor node is about 35 €, significantly lower compared to similar solutions present on the market. Considering a two-hectare field (as described in Figure 6), divided into two sectors of about one hectare each and placing a node every 500 m^2^ (extendable up to 2000 m^2^, but with rougher monitoring), 40 nodes (reducible up to 10) are required for covering the whole land; therefore, the cost of the WSN is about 1400 € (350 € for the solution with fewer nodes in any case sufficient to ensure a proper communication).

### 3.3. Description of Developed BLE Sensor Tag for Monitoring the Product Conditions and Supporting Traceability

A BLE sensor tag was developed for monitoring the correct storage conditions of the agri-food products during the storing or transportation, in order to assess its freshness and integrity, but also to support the product traceability. The parameters selected for verifying the good storage conditions are the concentration of total volatile organic compounds (TVOCs), NH_3_ (ammonia) and CO_2_, the environmental temperature and humidity [60]. The TVOCs concentration summarizes contributions from some common gaseous species produced during the deterioration of food, such as CH₃CH₂OH (ethanol), C_2_H_4_ (ethylene), CS_2_ (carbon disulfide), etc. [54,56].

Also, the NH_3_ represents a good indicator of protein decomposition, thus its concentration can be used to determine meat freshness [65]. Instead, the CO_2_ monitoring can be used to control the quality of stored cereal and detect their spoilage. In particular, CO_2_ concentrations between 600 and 1.500 ppm indicate the onset of mold growth due to excessive moisture inside the stored material or moisture infiltration into the structure; instead, concentrations higher than 1500 ppm indicate a serious mold growth affecting the whole material or an infestation of insects inside the stored food [66]. The developed BLE smart tag, offering a multiparameter detection, is featured by great versatility, so allowing its application to different food typologies (e.g., vegetables, fruits, meat, fish, etc.), unlike other devices reported in the literature or present on the market which are tailored for a specific product typology.

The designed sensor tag uses the HM-10 BLE module (manufactured by Jinan Huamao Technology co, Jinan City, China) based on CC2541 Integrated Circuit (IC) (manufactured by Texas Instrument, Dallas, TX, USA), a system-on-chip (SoC) combining a 2.4 GHz BLE transceiver, compliant to Bluetooth v4.0 protocol stack, with an 8051 MCU, featured by 128 KB flash memory, 8 KB RAM and several supporting features and peripherals (i.e., 12-bit ADC, 23 General-Purpose I/O Pins, Watchdog Timer). The wide range of peripherals and the integrated BLE transceiver, implementing the v4.1 BLE stack, along with the advanced PMU are the main reasons that justify the choice of CC2541 IC for the implementation of the BLE smart tag. It has three distinct low-power modalities (i.e., PMi, i = 1,2,3) with increasingly lower current consumption, making it ideal for portable devices, wearable applications and consumer electronics. The CC2541 was programmed using CC debugger and programmer, employing IAR Workbench for 8051 MCU.

The HM-10 module was set as iBeacon for supporting the product traceability in the whole supply chain; during the sleep period of the BLE tag, an advertising time interval of 546.25 ms was set for the iBeacon modality and the broadcasted packet universally unique identifier (UUID), major, minor, Tx power) was used to identify the food container where the BLE sensor tag was applied. Obviously, this tracking system aims to trace the agglomerations of food products with medium-large dimensions (pallets, bins, boxes, etc.) and not for consumer-level tracking. The BLE tag can be reused a large number of times, once the container is collected empty from the food shop or wholesaler by the farm. In addition, the tag is applied to the inner surface by two screws, thus it can be easily removed and applied to another container, in case of damage of the latter. For the intended use, since the BLE smart tag can be reused several times, the economic impact related to the purchase of the device is greatly reduced. Further details about the operation of the developed tracking system are reported in the following Section 5.

**Figure 10:**
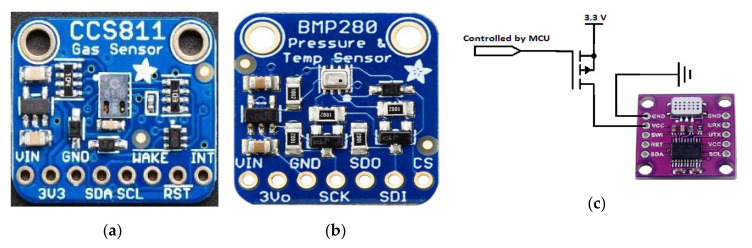
(**a**) CCS811 breakout board for detecting total volatile organic compounds (TVOCs) and eCO_2_ concentration; (**b**) BPM280 breakout board used for monitoring environmental temperature and humidity; (**c**) MiCS-5914 breakout board for detecting the ammonia concentration.

**Figure 11:**
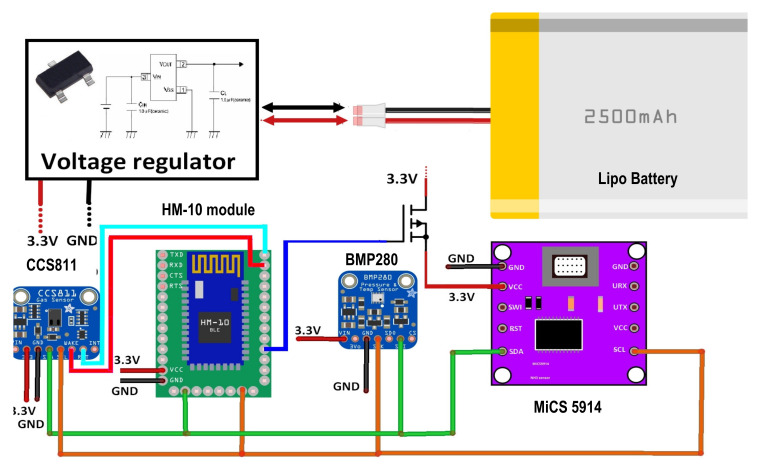
Schematic representation of the connections between the different components constituting the BLE smart tag.

The sensor tag is equipped with a CCS811 digital gas sensor (manufactured by AMS, Premstetten, Austria), an ultra-low-power metal oxide (MOX) gas sensor able to detect the TVOCs and equivalent CO_2_ (eCO_2_) concentrations for air quality monitoring applications. The measurements are obtained by means of intelligent algorithms able to process raw data related to current and voltage values provided by the MOX sensor and acquired by an integrated ADC, to then be made available to a host MCU using the I^2^C interface. Furthermore, the sensor has 3 low-power modes optimized for extending the autonomy in portable applications. The wide detection range (0 ÷ 1187 ppb for TVOC and 400 ÷ 8192 ppm for CO_2_), the high sensitivity, and the low power consumption make the CCS811 sensor ideal for the considered application, given also its high robustness to the external agents (high humidity and temperature, as well as presence of dust). The CCS811-based breakout board (manufactured by Adafruit Ind., New York, NY, USA, Figure 10a) was employed, modified to reduce the power consumption with the removal of the built-in 3.3 V voltage regulator (model MIC5225-3.3, manufactured by Microchip Technology, Chandler, AZ, USA), since the sensor was fed directly by the 3.3 V voltage regulator (model XC6206-3.3 with a lower quiescent current of only 25 µA in idle mode, manufactured by Torex Semiconductor, Tokyo, Japan) included in the power supply section of the BLE tag (Figure 11).

In addition, the sensor tag includes a BMP280 digital temperature and humidity sensor (manufactured by Bosch Sensortec, Reutlingen, Germany), featured by 300–1100 hPa pressure range and −40–+85 °C temperature range; it is based on a piezo-resistive pressure sensor combined with an integrated mixed-signal conditioning and acquisition section, then providing the conversion results through a digital interface (serial peripheral interface—SPI or inter-integrated circuit—I^2^C) (Figure 10b). The small dimension and the high number of the functionalities of BMP280 sensor allowed us to significantly reduce the overall size of the realized BLE tag, maintaining at the same time very low its power consumption. Three different operating modalities are available, to optimize the power consumption in low-power applications. To detect the ammonia concentration, a Micro-Electro-Mechanical System (MEMS) MOX gas sensor (model MiCS-5914, manufactured by Sensortech, Inc., Oxnard, CA, USA) was employed, featured by a 1–500 ppm detection range, a sensing resistance in the air between 10 KΩ and 1500 KΩ and sensitivity factor (R_air_/R_1 ppm_) between 1.5 and 15. A MiCS-5914-based breakout board (manufactured by Feng Tai Co., Hong Kong, China), equipped with an acquisition and transmission section based on the STM32 MCU (manufactured by ST Microelectronics Co., Geneva, Switzerland), was interfaced with the HM10 board by I^2^C serial communication (Figure 10c). The built-in 93 Ω pull-down resistor in series to the internal heater of the MOX sensor was substituted with a 47 Ω resistor, to power supply the board with 3.3 V and still ensuring the right value (2.2 V) of the heater voltage; furthermore, a high-side channel-P metal-oxide-semiconductor field-effect transistor (PMOSFET) switch was added to turn off the sensor, to reduce the power consumption when the sensor tag is in the idle mode.

The three sensors were interfaced through the I^2^C bus with the CC2541 TWI (two-wire interface) module corresponding to the pins 15 (serial data—SDA) and 20 (serial clock—SCL) of the HM-10 board, placing two 4.7 KΩ pull-up resistors on the two lines. In addition, the CCS811 sensor was kept in sleep condition when no measurement was required, thus reducing the current absorbed by the sensor (i.e., only 19 µA with a 1.8 V power supply); the sensor was woken up by putting down the nWAKE line (low active) through a GPIO (i.e., PIO11 pin) of the HM-10 board and then setting the third measurement modality (called Mode 3) in the MEAS_MODE register; after, 10 min have to be expected before the measurement. Similarly, also the ammonia sensor (MiCS-5914) was turned on 10 min before the reading, so allowing the sensor to reach the optimal operative temperature. Such time interval was determined through different experimental tests carried out on the two gas sensors (for TVOC and CO_2_ and NH_3,_ respectively), by comparing the obtained measures with those provided by two commercial gas detectors, namely a TVOC and CO_2_ combined detector (model ZN-202S, manufactured by Air Ae Steward, Inc., Shenzhen, China) and another for Ammonia (model WASO-XM, manufactured Hunan Gri Instrument Co., Hunan, China).

The power supply section is based on the XC6206-3.3 step-down voltage regulator for adapting the voltage provided by the 2500 mAh Lipo battery (model LIPO785060 manufactured by Pkcell battery Co., Shenzhen, China) to 3.3 V required by all the devices included in the BLE tag (Figure 11). The power consumption analysis of the BLE tag prototype is reported in the Section 4.2, as well as the description of the proposed tracking and tracing system based on the BLE smart tags and the mobile application is detailed in the Section 5.1.

The cost of the developed BLE smart tag is low, about 25–30 € including all sections, despite its superior functionalities compared to other competitor devices present on the market, which usually monitor only a single quantity (e.g., temperature or a single gas species). However, this cost is easily depreciable, given that the device is reusable for a long time, as previously described.

## 4. Results

### 4.1. Functional Testing and Power Consumption Analysis of Designed WSN Node

The developed WSN was tested in order to determine the effective power consumption of each node typology and thus establish the energy autonomy of the employed power supplying device. Specifically, the power consumption of a sensor node for different operating modes and scenarios are below reported. As described above, the original ESP8266 NodeMCU voltage regulator (model AMS1117-3.3) was replaced with a more efficient one (model MCP1825S-3302E) in terms of quiescent current and dropout voltage to reduce the node power consumption.

Power consumption is an essential factor in the performance and life of WSN. To analyze network operation, we take into account that each node sends its name, an ID message that identifies the number of data packets sent by each node, the Wi-Fi signal strength in the transmission and the sending time. This work emphasizes energy consumption due to the impact of Wi-Fi and BLE technologies. While interesting, other performance metrics, as the communication statistics, are not considered in this analysis, but are expected to be proposed in future work. Some of these parameters are related to the number of packets, where all the messages could be sorted by node and number of sent messages in order to obtain the lost messages in the network. With this arrangement, the messages that were not labeled as consecutive numbers are searched to verify that there was no jump. In addition, the nodes have timers that control the losses or delays and sliding windows for the flow control in the sending of data. In addition, since the network keeps all the nodes synchronized, it is enough to subtract the reception time and the emission time to remove the latency in the reception of messages. It is interesting to note that the nodes continue to send the message numbers without interruption, demonstrating a high network capacity against failures.

**Figure 12:**
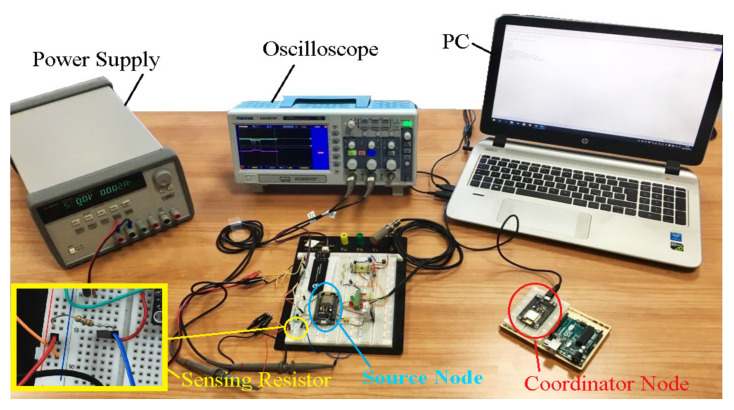
Experimental setup to determine sensor node power consumption in the different functioning modes, namely coordinator node searching-data acquiring and transmission–deep sleep mode.

The experimental setup for power consumptions analysis is depicted in Figure 12; the sensor node was fed with 3.7 V, provided by a power supply generator (model E3631A, manufactured by Agilent Technologies, Santa Clara, CA, USA), directly applied to the voltage regulator input, voltage value chosen to reproduce the real use of the node fed by the LiPo battery or 90 F super-capacitor. A digital oscilloscope (model DSO5072P, manufactured by Hantek, Shandong, China) was used to acquire the voltage drop waveforms across a 4 Ω sensing resistor in series with node power supply line, for determining absorbed current values during the different operating phases (inset of Figure 12). In order to accurately measure the very low absorbed current during deep sleep modality, also a digital multimeter (model GDM8351, manufactured by Gwinstek, Taipei, Taiwan, with 1 µV resolution) was placed in parallel to the 4 Ω sensing resistor. In addition, from the acquired signals, the time duration of each node operating phase is determined to calculate the related energy consumption.

**Figure 13:**
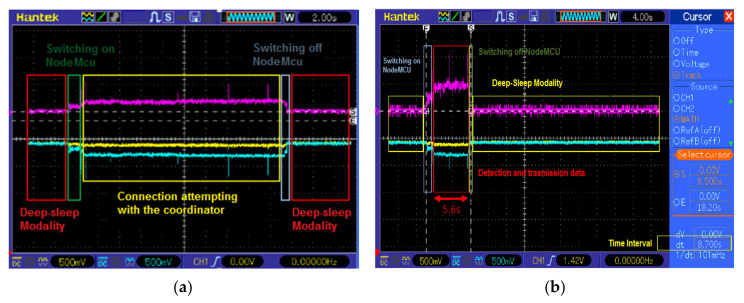
(**a**) Oscilloscope traces acquired during unsuccessful connection attempt of the sensor node with coordinator node; after 20 s, the node returns in deep sleep mode to reduce the power consumption; (**b**) oscilloscope traces acquired during a successful connection of the sensor node with coordinator node; only 5.6 s were required to complete the data detection and transmission phases.

In Figure 13a, the oscilloscope traces related to the 4 Ω sensing resistor terminals (yellow and light blue lines) and to their difference (purple) are reported, thus being able to easily distinguish each node functioning mode, namely the wake up (green box), unsuccessful data transmission to an unavailable coordinator node (yellow box) and subsequent return to the deep sleep modality (NodeMcu switching off, red box). As above described, the sensor node tries to establish a connection with the coordinator node for a time duration of 20 s (as depicted in the flowchart of Figure 9), otherwise, it returns in deep sleep mode to reduce its power consumption (Figure 13a). The absorbed current values from the sensor node, during all the operating phases, are then determined.

In Figure 13b, the same signals of Figure 13a are reported when a connection was established between the sensor node and the corresponding coordinator. At first, the sensor node wakes up from deep sleep mode, switches on the connected sensors to acquire interest data and then establishes a connection with the coordinator node; in this phase, the data are acquired by sensors and sent to the coordinator node (red box). Finally, the ESP8266 NodeMCU switches off the connected sensors (green box) and returns in deep sleep mode (yellow box) for 30 min, as described in Figure 9. Similarly, the current values and time duration of different operative phases were determined; the time interval needed to complete the acquisition and transmission phases is equal to 8.7 s (Figure 13b), while a 5.6-s-long time is required for the only data transmission. The time interval required to establish the connection varies over the different operating cycles according to the availability of coordinator node; however, a 3.1 s mean value was determined based on the several carried out experimental tests.

Given the above-reported data relative to both time durations and current values during the different sensor node operating phases, its energy consumptions can be calculated. Because a 4 Ω sensing resistor was used, the voltage drop across it and the corresponding absorbed current associated with each operative phase, are following reported in Table 1.

If the sensor node is fed by 3.7 V power supply voltage (provided by the 100 mAh LiPo battery or 90 F super-capacitor) applied to the ESP8266 NodeMCU voltage regulator input pin, the energy consumption estimate of the whole sensor node operating cycle is given by the following relation:

(1) Estimated Energy Consumption=1800 s×0.067 mA×3.7V+3.1 s×20.156 mA×3.7 V+5.6 s×35.125 mA×3.7 V=1.405 J=390.333 μWh,

Given the current values of each source node operative phase, the voltage drops on the 90 F feeding super-capacitor (charged to 5 V by placing on the LTC3105-based conditioning board the jumper for enabling the 5 V output voltage) were calculated as following reported:

- Deep-sleep mode:
  (2) $$Q_{1}=0.067\;\mathrm{mA}\times 1800\;s=0.1206\;C\to \Delta V_{1}=\frac{Q_{1}}{C}=\frac{0.1206\;C}{90\;F}=1.340\;\mathrm{mV},$$
- Coordinator node searching mode:
  (3) $$Q_{2}=20.156\;\mathrm{mA}\times 3.1\;s=0.0625\;C\to \Delta V_{2}=\frac{Q_{2}}{C}=\frac{0.0625\;C}{90\;F}=0.694\;\mathrm{mV},$$
- Acquisition and data transmission mode:
  (4) $$Q_{3}=35.125\;\mathrm{mA}\times 5.6\;s=0.1967\;C\to \Delta V_{3}=\frac{Q_{3}}{C}=\frac{0.1967\;C}{90\;F}=2.185\;\mathrm{mV},$$

Therefore, the overall voltage drop for a single sensor node operating cycle is given by:

(5) $$\Delta V=\Delta V_{1}+\Delta V_{2}+\Delta V_{3}=1.340\;\mathrm{mV}+0.694\;\mathrm{mV}+2.185\;\mathrm{mV}=4.219\;\mathrm{mV},$$

Considering that the sensor node carries out 48 cycles for each day and that its minimum supply voltage allowed is 3.2 V, then its energy autonomy is determined by the following relation:

(6) $$Autonomy\;\left[\mathrm{gg}\right]=\frac{5\;V-V_{in,\min}}{48\times \Delta V}=\frac{\left(5-3.2\right)\;V}{48\times 4.219\times 10^{-3}V}=8.88\;\mathrm{gg},$$

Therefore, a sensor node energy autonomy of almost 9 days was obtained, in the condition in which the energy contribution from the solar panel for recharging the 90 F super-capacitor storage device is totally absent due to the bad weather conditions (unrealistic eventuality).

**Figure 14:**
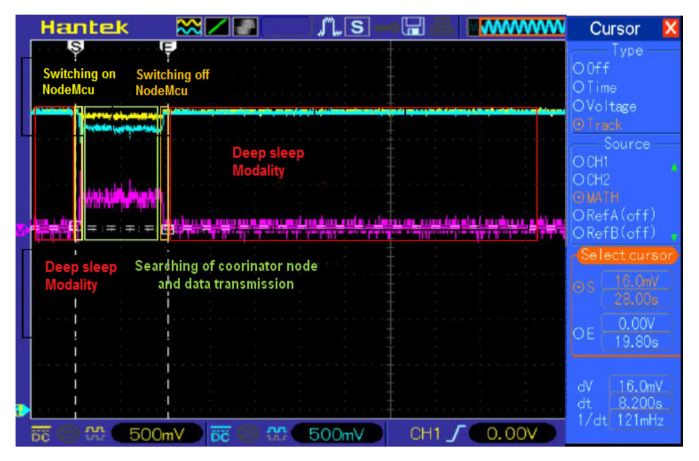
Oscilloscope traces related to the voltage drop on the 4 Ω sensing resistor (yellow and light blue) during a successful connection of the sensor node with the coordinator node. The time duration to complete the acquisition and transmission phase is equal to 8.2 s.

Afterwards, the sensor node was power supplied by a 100 mAh single cell LiPo battery, recharged by the solar energy harvesting system (by placing the jumper correspondent to the 4.1 V output voltage on LTC3105-based conditioning board); through the same experimental setup of Figure 12, the voltage drop across the 4 Ω sensing resistor during the different sensor node operating modalities was acquired by the digital multimeter. Similarly, the absorbed current values and the respective time durations were determined (Figure 14) and thus the charge amount required by the sensor node during the different operating phases calculated, as following reported:

- Deep-sleep mode:
  (7) $$I_{1}=\frac{\Delta V_{1}}{R_{sens}}=\frac{0.273\;\mathrm{mV}}{4\;\Omega }=0.068\;\mathrm{mA}\to Q_{1}=I_{1}\times t_{1}=0.068\;\mathrm{mA}\times 1800\;s=\;0.122\;C\;\left(0.034\;\mathrm{mAh}\right),$$
- Coordinator node searching mode:
  (8) $$I_{2}=\frac{\Delta V_{2}}{R_{sens}}=\frac{83.928\;\mathrm{mV}}{4\;\Omega }=20.982\;\mathrm{mA}\to Q_{2}=I_{2}\times t_{2}=20.982\;\mathrm{mA}\times 3.1\;s=\;0.065\;C\;\left(0.018\;\mathrm{mAh}\right),$$
- Acquisition and data transmission mode:
  (9) $$I_{3}=\frac{\Delta V_{3}}{R_{sens}}=\frac{149.012\;\mathrm{mV}}{4\;\Omega }=37.253\;\mathrm{mA}\to Q_{3}=I_{3}\times t_{3}=37.253\;\mathrm{mA}\times 5.6\;s=\;0.208\;C\;\left(0.058\;\mathrm{mAh}\right),$$

As can be noted, the current and charge values obtained are very similar to those previously reported. Therefore, the electric charge needed to complete a single operating cycle is given by:

(10) $$Q_{TOT}=Q_{1}+Q_{2}+Q_{3}=0.034\;\mathrm{mAh}+0.018\;\mathrm{mAh}+0.058\;\mathrm{mAh}=0.110\;\mathrm{mAh},$$

Given that the system performs 48 operating cycles per day and assuming a 30% LiPo battery discharge margin, the sensor node autonomy could be expressed by the following relation:

(11) $$Autonomy\;\left[\mathrm{gg}\right]=\frac{0.7\times Battery\;Capacity\;\left[\mathrm{mAh}\right]}{48\times Q_{TOT}\;\left[mAh\right]}=\frac{0.7\times 100\;mAh}{48\times 0.11\;\mathrm{mAh}}=13.26\;\mathrm{gg},$$

A 70% discharge margin was supposed because within that limit the LiPo battery can ensure the right supply voltage for the sensor node (minimum value = 3.2 V), below that state of charge then the battery voltage goes down suddenly. Therefore, a sensor node energy autonomy greater than 13 days was obtained with the developed system, in conditions of absence or limited availability of solar energy to recharge the used storage device. The different discharge profile of LiPo batteries respect to super-capacitors allows a greater sensor node energy autonomy, thus the LiPo batteries must be considered more appropriate for the considered application.

### 4.2. Operation Modalities and Power Characterization of Developed BLE Smart Tag

**Figure 15:**
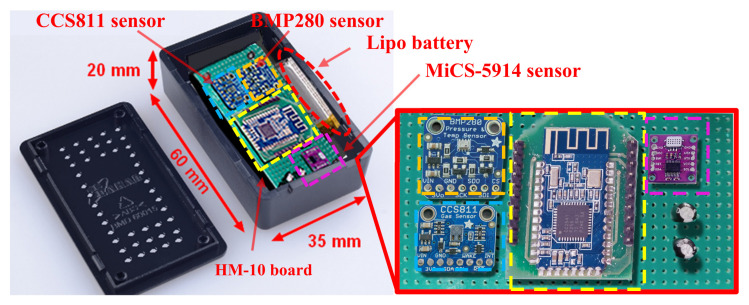
The prototype of the developed BLE smart tag placed inside a plastic box with a perforated cover.

The prototype of the BLE smart tag was realized by placing the sensing breakout boards on a prototyping board and realizing the connections on its back (Figure 15); to simplify the wiring, the HM-10 module was soldered on an adapting board in order to expose all its pins, making easy the connection with the programmer. In addition, the voltage regulation section, based on the XC6206-3.3 IC, was placed on the back of the prototyping board, as well as the LiPo battery on the bottom of the plastic box, under the electronic boards; for sake of clarity, the LiPo battery is shown in Figure 15, besides to the board. All the mentioned components were positioned inside a plastic box, with a perforated cover to allow the right detection of parameters inside the container where the BLE sensor tag is installed.

**Figure 16:**
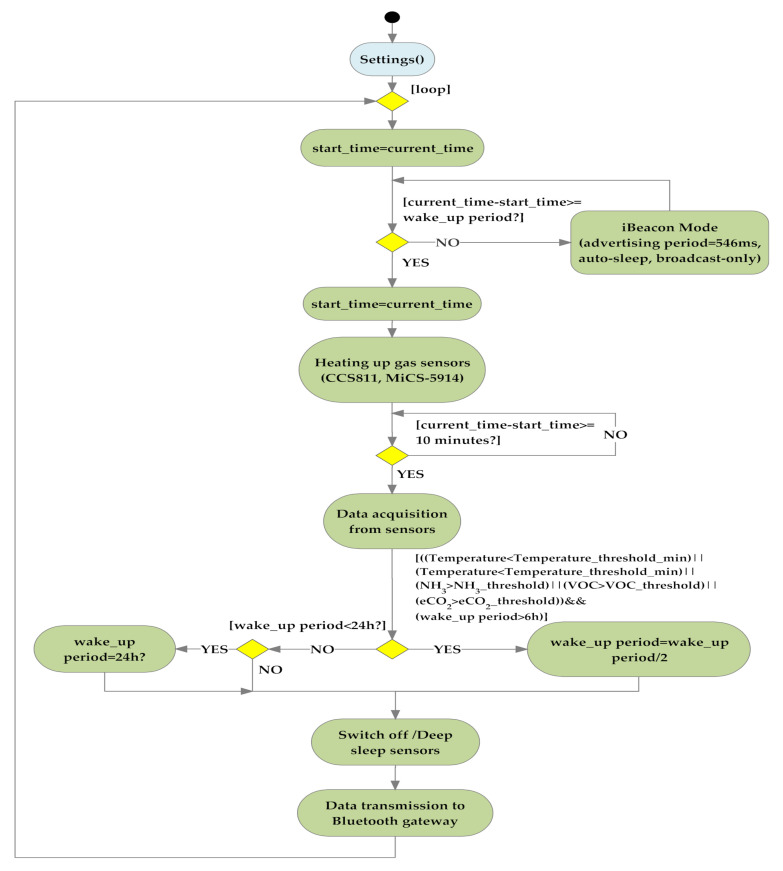
Flowchart related to the operation modality of developed Bluetooth low energy (BLE) smart tag placed inside the container where the agri-food products are stored.

The operation modality of the developed BLE smart tag is described in the flowchart reported in Figure 16; at first, the setting of different interfaces and registers of the CC2541 IC (microcontroller on board to the HM-10 control module) has to be defined, to allow connection with the employed sensors. Afterward, for a time interval equals to the wake_up period (default value 24 h), the HM-10 module is set in the iBeacon modality, broadcasting an advertising packet every 546.25 ms (time interval between the advertising packets, as detailed in Figure 18a) by exploiting the halSleep() and osal_set_event() methods of the BLE v1.4.1 stack; when the module doesn’t transmit any advertising packets, the HM-10 module brings itself in PM3 low power modality, so allowing a great reduction of the power consumption. Once the sleep timer overflows the wake_up time interval (default value 24 h), the HM-10 module is switched to the active mode, thus waking up the CCS811 sensor and switching on, by the CMOS switch, the MiCS-5914 sensor for 10 min allowing them to reach their operative temperature; as previously described, such time interval was experimentally determined, as the minimum value required by the two sensors for reaching their operative temperature. After this time interval, also the BMP280 temperature and moisture sensor is woken up and then all the data are acquired from the sensors and elaborated for extracting the aforementioned parameters (i.e., gas concentrations, temperature and humidity, Figure 16). Afterward, each parameter is compared with specific thresholds, chosen as a function of the stored agri-food product typology, since each food typology is featured by a storage temperature and specific gas types that indicate the food spoilage, as reported in the literature [59,67,68] (Figure 16); besides, such threshold values depend on the shape and size of the food container, on the positioning into the container of the tag respect to the product level, as well as on the presence of ventilation, namely if the food package is sealed or open. The threshold values can be set or modified by reprogramming the BLE smart tag, every time that the agri-food product stored in the container is changed.

**Figure 17:**
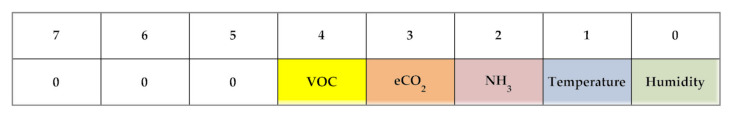
Structure of the warning byte transmitted from the BLE tag to signal an alarm condition related to the acquired parameters.

If abnormal parameter values are detected, the wake_up period is halved compared to the previous one, up to a minimum value of 6 h; afterward, only if no monitored parameter is beyond the preset limits, the wake_up period is reset to 24 h (Figure 16). Following, the BLE tag enables the deep sleep mode for the BMP280 and CCS811 sensors and turns off the MiCS-5914 sensor and then it establishes the connection with the Bluetooth gateway, towards which transmits the acquired data along with a warning byte, which indicates if a certain parameter has assumed an abnormal value (alarm condition). Specifically, 5 bits of the warning byte are associated with the 5 parameters acquired by the BLE tag, arranged as depicted in Figure 17 (each bit set to “1” logic in case of an alarm condition). Finally, the operating cycle above described starts again with the BLE smart tag set in the iBeacon modality, with the same setting previously reported, for the time interval (24 h or 12 h or 6 h), as determined in the previous cycle.

During both iBeacon modality and data transmission phase of the BLE smart tag, the transmitted power was set to 0 dBm by using hciStatus_t HCI_EXT_SetTxPowerCmd() method, in order to comply with the detection range requirements described in the following Section 5.1.

**Figure 18:**
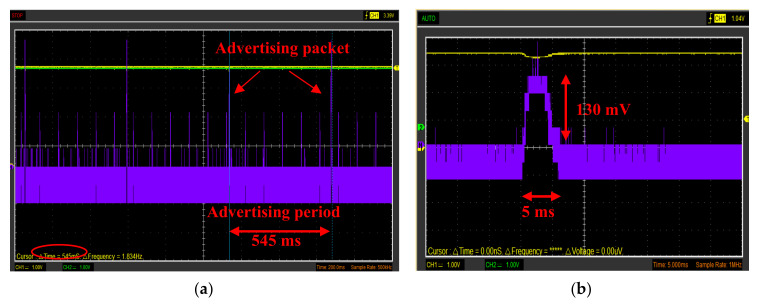
(**a**) Oscilloscope traces acquired during the iBeacon modality of the BLE smart tag; every 546.25 ms an advertising packet is transmitted, indicated by a peak of the voltage drop on the sensing resistor; (**b**) the peak voltage drop is equal to 130 mV, corresponding to an absorbed current value of 13 mA.

Afterward, the BLE smart tag was tested for determining its overall power consumption, thus establishing the energy autonomy. A GDM8351-model digital multimeter was placed in series to the power supply line of the BLE tag, for acquiring the current values with a 100 ms sampling rate to determine the power consumption for each operative phase. In Table 2, the measured current values are summarized, along with the time duration of each phase determined by placing a 10-Ω sensing resistor in series to the power supply line and monitoring, with a DSO5072P-model digital oscilloscope, the voltage drop on the sensing resistor. The measures were carried out by considering a 24 h wake_up period and the time durations reported in Table 2 refer to a single operative cycle of the BLE tag (set of phases outlined in the flowchart of Figure 16). In addition, in Figure 18a the oscilloscope traces related to the 10-Ω sensing resistor terminals (yellow and green lines) and their difference (purple) are reported, during the iBeacon modality of BLE smart tag; as it can be noted from Figure 18b, a 130 mV peak value for the 10-Ω drop voltage was measured during the packet transmission (time duration of 5 ms), corresponding to a 13 mA absorbed current. As can be noted in Figure 18a, the time interval between the iBeacon packet transmissions, represented by the current absorption peaks, is equal to about 545 ms, time interval previously set during the setting phase of the CC2541 module (Figure 16). Thanks to the firmware strategies implemented in the flowchart of Figure 16 for the power consumption minimization of the different modules, a very long lifetime (more than 5 months) of the employed LiPo battery was obtained, as reported below.

Once measured the current values for each BLE tag operative phase, then the overall charge required to carry out an operating cycle was calculated as follows:

- iBeacon phase:
  (12) $$Q_{1}=0.237\;\mathrm{mA}\times 24\;h=5.688\;\mathrm{mAh},$$
- Heating phase of the gas sensors:
  (13) $$Q_{2}=28.287\;\mathrm{mA}\times \frac{600}{3600}\;h=4.714\;\mathrm{mAh},$$
- Acquisition and transmission phase:
  (14) $$Q_{3}=8.675\;\mathrm{mA}\times \frac{1.426}{3600}\;h=0.003\;\mathrm{mAh},$$

The overall charge required for a single operative cycle of the BLE tag is given by the following formula:

(15) $$Q_{TOT}=Q_{1}+Q_{2}+Q_{3}=5.688\;\mathrm{mAh}+4.714\;\mathrm{mAh}+0.003\;\mathrm{mAh}=10.405\;\mathrm{mAh},$$

Following, assuming a 30% LiPo battery (with a 2500 mAh capacity) discharge margin, the energy autonomy of the BLE smart tag was determined:

(16) $$Autonomy\;\left[\mathrm{gg}\right]=\frac{0.7\times Battery\;Capacity\;\left[\mathrm{mAh}\right]}{Q_{TOT}\;\left[\mathrm{mAh}\right]}=\frac{0.7\times 2500\;\mathrm{mAh}}{10.405\;\mathrm{mAh}}=168.20\;\mathrm{gg},$$

Therefore, an energy autonomy greater than 168 days (i.e., 5.6 months) was obtained for the developed BLE sensor tag, fully satisfactory for the considered application.

## 5. Discussion

As discussed above, the smart making-decisions section of developed IoT-based farm facility is the software platform, enabling the control and management of all its functionalities and offering to the customers some value-added services related to the traceability of agricultural products; specifically, the platform includes the so-named Campaign Registry application, able to monitor and establish, in an optimized way, the fertigation treatments for each monitored land area on the basis of the following inputs and agronomic models:

- WSN data related to environmental, soil and crops parameters;
- User inputs, namely data entered, through the dedicated “Farm App” installed on the smartphone/tablet, by the farmer or agronomist;
- Data obtained by reliable weather stations;
- Purchase orders, namely information able to influence the growth or production of a specific crop if the market requires greater or lower production levels.

The cloud database is managed by professional users (as informatics or agronomists) in order to constantly update the set of information (temperatures, soil data, fertilizer types, fertigation periods, weather conditions, crops growth status), taking into account any change in the state of the land over time, for optimizing the quality and yield of cultivated products and, at the same time, preserving the soil wellbeing with consequent advantages for the ecosystems. Furthermore, the agronomist can update the data into the on-cloud database on the basis of the on-field observations related to the crop phenological development. In addition, he can also check the fertigation system correct operation by sending a “status query” through the smartphone app to the remote management platform as well as he can identify a land portion with his smartphone by reading the QRcode, placed near each portion of the cultivated land, in order to view or update the related information contained in the “Campaign Register” application of the software platform and finally he can request the execution of a specific treatment of fertigation or against pests, on the considered land portion bypassing the remote automated management.

The developed software platform automatically stores detailed information concerning the treatments performed on followed cultivations during the whole growth period, allowing the customers to verify, through a suitable user interface, the treatments carried out on the considered agricultural products, and thus their quality, but also to place a purchase order. Through the developed platform, the consumers can monitor the whole supply chain of the agri-food products, obtaining information concerning the freshness and genuineness of the agricultural product, so enabling additional revenues to the farmer. In addition, the data and parameters of the general agronomic database can be monitored and updated by the farmer or agronomist, as well as they can manage the execution of specific treatments through the developed platform. The customers can access only to the information concerning performed treatments and to product traceability [69]. The data derived from the agronomic models, crucial for the fertigation system operation, are stored both in the on-cloud database and in the local one, data concerning the nutritional needs of each considered crop typology as function the seasonal period, growth phase and specific environmental/geographical/meteorological conditions.

In fact, the possible system operation modes are two, namely:

- Bottom-Up modality: the master is a stand-alone component (PC of the solar-powered fertigation system) where the main operating activities are performed, stored and synchronized with the on-cloud platform (remote server). In this way, the service is guaranteed even if the Internet connection is absent or not always guaranteed.
- Top-Down modality: the master is the on-cloud architecture that manages and commands the needed operations to be performed by the solar fertigation system; moreover the farmer can remotely set the fertigation cycles and land sections, where to carry out the farming treatments.

The fertigation system has two user levels, namely:

- Level 1—System Management: the authorized user can access to each software section and application as a master user, can create a lower-level user (farmer/customer), visualize and remotely control each installed fertigation system and manage all their operations. Further, he can update the general agronomic database by adding further crops to be cultivated or modifying the parameters and calculation algorithms;
- Level 2—Farmer or Customer: he is associated with a single fertigation system, can set the crops in the different areas of the land (only the farmer), control automatically or manually the irrigation or fertigation cycles (only the farmer), view the history of crops parameters and certification reports of the carried out treatments.

The developed application comprises a traceability system of cultivated crops, named *Customer Free Campaign Register*, an open-source tool, connected to the general agronomic database, by which the customer can verify the farming processes and quality standards, even before arranging the purchase order, so providing an added value for the farmer. The software platform allows enabled users to remotely interact with the farm management system, for example, to check the historical register of the different carried out agricultural processes or to update the agronomic database based on observations directly on cultivated land related to crop growth.

**Figure 19:**
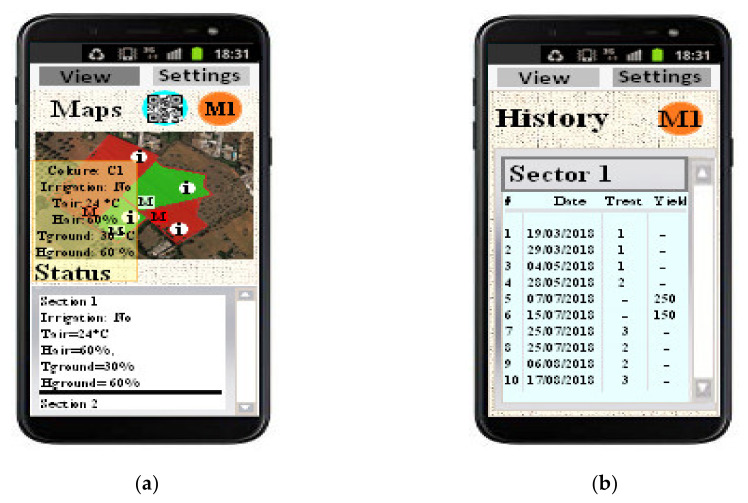
Screenshots of the realized application: “view section” where the farmer (user M1) has a complete overview of the cultivated crops (**a**) and “Data history section” through which the farmer or customer monitor crops’ parameters and the performed treatments for growth optimization (**b**).

In order to highlight the functionalities and utility of developed smartphone application for the farmers or customers, following some screenshots are shown and described. In Figure 19a, the “view section” is shown from which the farmer (user M1) can check the irrigated sectors (green color); clicking on “*Status*” button, the enabled user can control soil and crops parameters detected by the WSN whereas with the M-button he can manually command a specific process for the considered land sector bypassing what established by the DSS on the basis of the agronomic models. In Figure 19b, the “Data history section” for *sector 1* is shown with the chronology of the WSN data and performed treatments; to such section, both the farmer and customer users can access from their account.

Finally, to verify the correct functioning of the developed IoT-oriented farm management facility constituted by the sensor nodes installed in the cultivated ground and by the cloud software platform, different measurement campaigns were carried out actually on cultivated lands and the obtained results have certified the correct functioning of the hardware and software sections.

### 5.1. Proposed Tracking and Tracing System for Monitoring the Storage Conditions of Agri-Food Products and Real Tests of the Designed BLE Smart Tag

**Figure 20:**
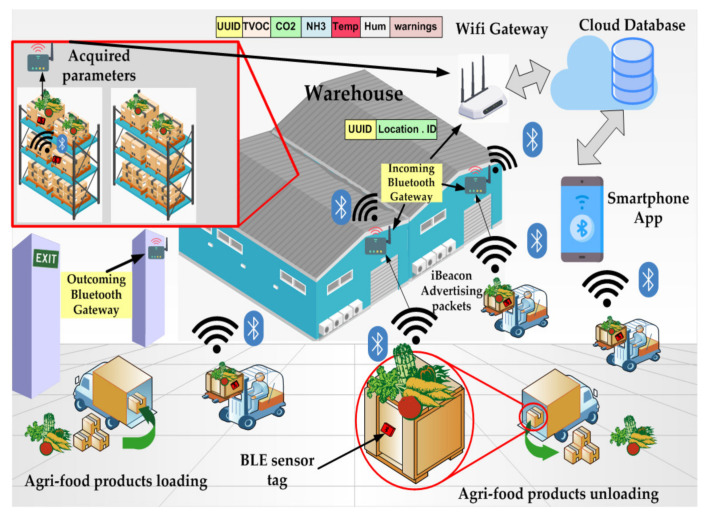
Schematic representation of the proposed tracking and tracing system based on the BLE smart tags.

As previously described, the proposed farm management and control system includes a traceability system of the agri-food products along the whole SC able to monitor and share the main indicators for determining the storing and transport conditions. This system is based on the BLE smart tags, with the structure described in the Section 3.3, placed into the containers where the agri-food products are stored. Once the products arrive at a storage warehouse, the UUID of newly arrived containers are detected by the Bluetooth gateway (called “Incoming Bluetooth Gateway”, in Figure 20) located near the warehouse unloading area; when a new UUID is found, the incoming gateway updates, through a Wi-Fi gateway, the new container location in the relative record, associated with the detected UUID, in the cloud database (i.e., an Azure SQL database).

A commercial Bluetooth gateway (model BTP104-SS, manufactured by Jinou, Chongqing, China) was employed for carrying out detection and communication tests of the BLE smart tags with the on-cloud platform (as represented in the scenario of Figure 20); by setting the transmitted power level of each BLE tag to 0 dBm, a detection range of 12 m was obtained, particularly, in the condition in which the tagged containers are stacked for their transportation, reaching instead up to 21 m detection distance in the open field condition. Therefore, a gateway can be located at the warehouse entrance for detecting the incoming containers, whereas inside the warehouse the gateways have to be regularly distributed (at a distance of 12 m from each other) for receiving the data transmitted by the tags (Figure 20).

After the detection of a new container, the Bluetooth gateway sends to the remote database a packet containing its identifier (i.e., the UUID) along with a code related to the new storage warehouse (Figure 20). The UUID is the key for all the operations carried out in the database, namely to insert, select, update, delete and call an item. Afterward, the food products are stored in the warehouse and the BLE smart tags, placed into the containers, periodically send the acquired parameters, suitably arranged in data packets, to the Bluetooth gateways located inside the warehouse. Specifically, a 27 bytes packet is transmitted by each BLE tag, with the first 16 bytes represent its UUID, 6 bytes are related to the TVOC, CO_2_ and NH_3_ concentrations and 2 bytes each for the environmental temperature and humidity (as depicted in Figure 20). Finally, the warning byte is added to the packet to signal anomalies of the monitored parameters, as described in the Section 4.2. The data related to a given container, then, are stored into a record of the on cloud database, related to the BLE tag UUID; when a container leaves the warehouse towards another one, it passes nearby another gateway, called “outcoming Bluetooth gateway”, which updates the database reporting its exit. The afore-described mechanism continues until the containers are delivered to the food shop or wholesaler, where also the empty tagged containers are picked up by the farmer to be following reused; the data stored in the database related to a given UUID are removed after the expiration date of the food product.

The user can assess the tracking and storage information of the agri-food products employing a mobile application designed by using the Android Studio 3.6.1 development environment.

**Figure 21:**
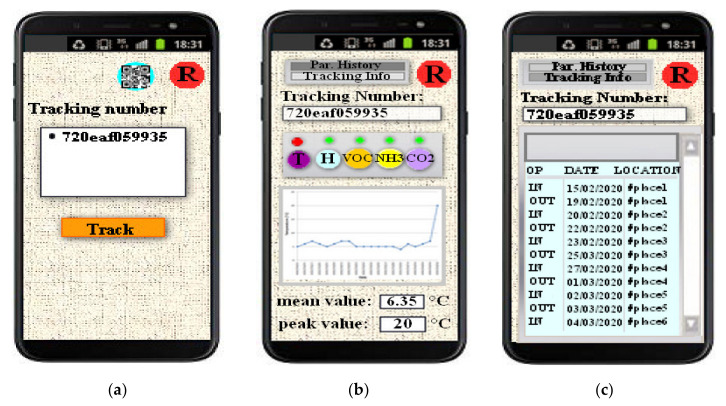
Application screenshots of the tracking and traceability system based on BLE tags. (**a**) logged user (R) inserts the container universally unique identifier (UUID); (**b**) “Par. History” section with shown the history of acquired parameters; (**c**) “Tracking Info” section where the customer checks the tracking information related to the entered UUID.

After the login, the user (indicated with R, Figure 21a) is directed into the main page of the application, where the container UUID can be entered typing the alphanumeric characters in the appropriate field and by pushing the “Track” button, the user is directed to the page related to the desired container (Figure 21a). Afterward, by pushing the “Par. History” button (Figure 21b), the user can verify the history of each monitored parameter pressing the button related to the desired quantity (i.e., T, H for humidity, VOC, NH_3_ and CO_2_); a visual indicator is added above each button to signal abnormal values of the corresponding parameter. In addition, the temporal trend of the selected parameter is shown (e.g., the temperature in Figure 21b) along with its mean and peak values.

By pushing the “Tracking Info” button, the user can monitor the tracking information of the considered container (Figure 21c); in fact, this page contains a table where are detailed all the movements of the container, updated over time by means of the tracking system above described.

Several tests were performed for validating the operation of the BLE tags and related tracking system; the BLE smart tag was placed inside a plastic box (with 50 cm × 40 cm × 40-cm dimension) covered by a perforated lid (30 holes with 0.6-cm diameter), containing 5 kg of oranges, leaving the box into a refrigerator set to a 5 °C target temperature for 20 days. On the 20th day, the plastic container was taken out of the freezer and left at room temperature (about 20–25 °C) for further 10 days. The tag was fixed by means of screws on the inner surface of the plastic container, 10 cm above the product level since, from preliminary tests, this position can ensure a good detection capability to the device, and, at the same time, avoid some false alarms due to local gas accumulation. However, the container dimension, shape and ventilation affect the response of the BLE smart tag, since these factors determine the gas concentration and distribution inside the container; therefore, a preliminary characterization of the BLE tag, before to be used for the specific container, is required to determine the threshold values for each gas species. For the case under consideration, the threshold values were determined based on visual and olfactory observations and set to 581 ppb for TVOC, 176 ppm for NH_3_ and 1647 for CO_2_.

If the concentration of a gas species exceeds the aforementioned threshold or the temperature and humidity values are outside the set intervals (0–10 °C and 30–70% for oranges, respectively), an alarm bit is set in the warning byte and sent to the software platform along with the acquired information. Besides the temporal trends, the mobile application shows the user the warning on the monitored quantities, as a visual indicator which turns red (from green) in case of alarms. For instance, in Figure 21b, the data related to the container with oranges are reported; once the container is removed from the refrigerator, the indicator related to the temperature (marked with T) changes from green to red.

**Figure 22:**
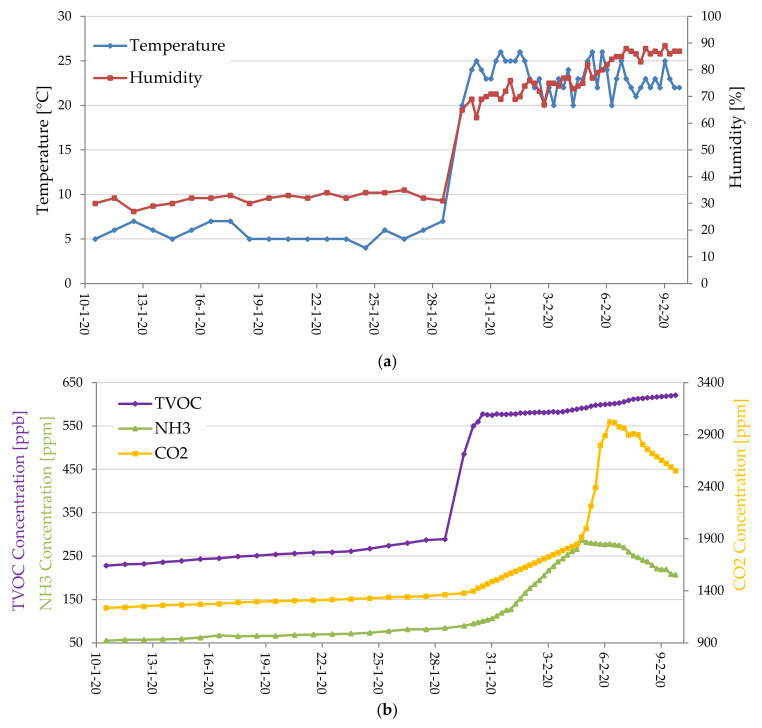
Temporal trends of the parameters detected by the BLE tag applied to the plastic box with 5 kg of oranges inside, during 30-day-long period. (**a**) Temperature and humidity; (**b**) TVOC, NH_3_ and CO_2_.

The temporal trends of the monitored parameters are shown in Figure 22; as it can be noted, the measurements are temporally closer from the 20th day, since the detected temperature is higher than the upper threshold (set to 10 °C) and thus the wake_up period is progressively reduced up to 6 h, as detailed in the Section 4.2 (Figure 22a). After removing the box from the refrigerator, TVOC values increased significantly due to the fastening spoiling process into the stored oranges [70,71]. In addition, the measurements showed an increase of CO_2_ and NH_3_ values throughout the measuring range, slow in the refrigerator and faster outside, reaching peak values of 3022 ppm and 287 ppm, respectively (corresponding to 5439.6 mg/m^3^ and 199.6 mg/m^3^). Following, a reduction of CO_2_ and NH_3_ values in the last days of the observation interval was observed, as already reported in the literature [70] (Figure 22b).

**Figure 23:**
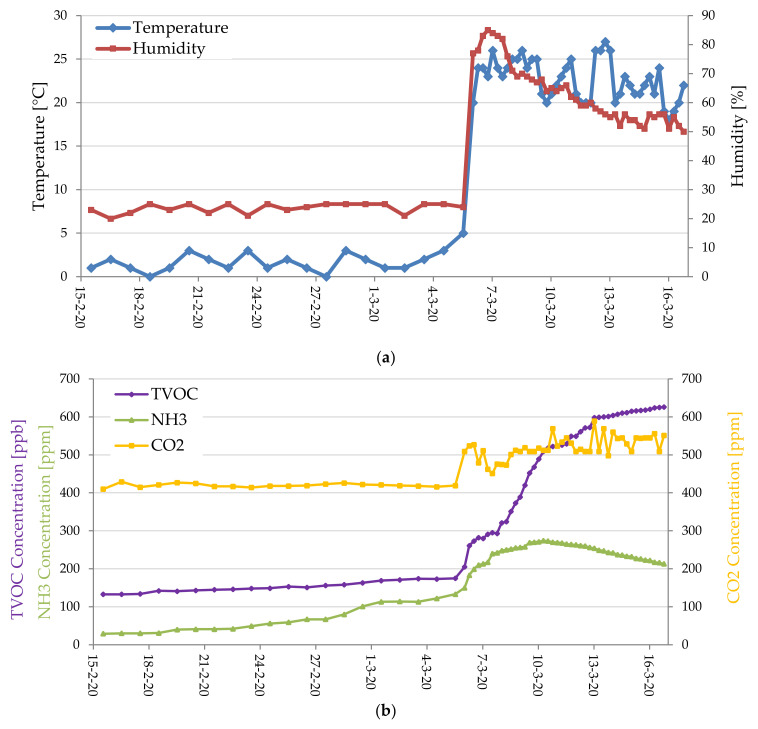
Temporal trends of the parameters detected by the BLE tag applied to the box with inside 500 g of codfish fillets during 30-day-long period. (**a**) Temperature and humidity; (**b**) TVOC, NH_3_ and CO_2_.

A similar test was carried out on 500 g of frozen codfish fillet placed inside a polystyrene box with 40 cm × 40 cm × 20 cm dimension, covered by a plastic wrap and leaving the container to 0 °C for 20 days; then the box is removed from the freezer and left uncovered to room temperature for other 10 days. The BLE tag was attached to the internal surface of the polystyrene box, 10 cm above the fish level, in order to monitor the storing conditions and the gas levels emitted by the codfish fillets; the related temporal trends, over the whole observation interval, are reported in Figure 23. Similarly, the BLE tag characterization for the container with fish inside was performed, to determine the threshold values, so obtaining 389 ppb and 256 ppm values for TVOC and NH_3_, respectively; besides, the temperature and humidity ranges were set to 0–10 °C and 20–70%. Thus, if a detected value isn’t included in the corresponding set range, an alarm message is sent to the software platform and reported on the mobile application by means of the visual indicator (turned red) related to the abnormal parameter (Figure 21b). In the first phase, the NH_3_ concentration increases slowly although in the refrigerator, since the enzymatic and bacteria growth decomposes the fish proteins even at a temperature of a few degrees [72], so producing nitrogen volatile compounds that indicate the spoiling of the fish. This trend has accelerated after the defrosting (from the 20th day) reaching a peak value of 274 ppm (i.e., 190.5 mg/m^3^) after about 4 days, then the NH_3_ concentration reduced indicating an advanced decomposition of fish proteins, as already reported in [73] (Figure 23b). A CO_2_ concentration near to the lower limit of the MiCS-5914 sensor (i.e., 400 ppm) was detected before the box left the freezer, values ascribable to the environmental concentration inside the box; in fact, once the box was extracted from the freezer and removed the plastic wrap, the CO_2_ concentration is increased slightly around the value of 500 ppm, due to the indoor environment. TVOC concentration, initially, grew slowly (up to 170 ppb) until the box was in the freezer, increasing then quickly when the box was extracted, reaching a maximum value of 626 ppb at the end of the observation interval (10 days after the defrosting).

### 5.2. On-Field Testing of the Developed WSN-Based Farm Management Facility in Real Scenarios

Afterward, different measurement campaigns were carried out on-field for testing the operation of all the fertigator sections, but also the proper integration of the software platform, implementing the agronomic database, with the developed WSN and the weather forecast; in this way, the benefits in terms of water savings and proper fertilizer usage were evaluated.

At first, the system was tested on a land of 1200 m^2^ cultivated with tomatoes, arranged in rows equipped with a drip irrigation system and divided into three sectors. In this case, a fertigation unit equipped with a 5000 L mixing tank and 3 hp pump (model HF-8B, manufactured by Pedrollo Spa, San Bonifacio, Italy) was employed. A WSN constituted by 6 sensor nodes, two for each sector, was installed with one of them acting as coordinator node. The considered land is featured by slightly clay soil (i.e., a high TAW—total available water value), whereas the tomato plants are in the development phase (20 days from the planting); specifically, the reported tests were carried out on 22 July 2019, during a week characterized by high environmental temperatures and humidity. In these conditions, the agronomic database scheduled a unique irrigation per day, lasting an hour and a half, corresponding to 1800 L water amount for each sector; in addition, the agronomic database established a fertigation process with 7.5 kg of N–P–K soluble fertilizer (with 9–30–22 ratios) dissolved into the water amount of a normal irrigation.

**Figure 24:**
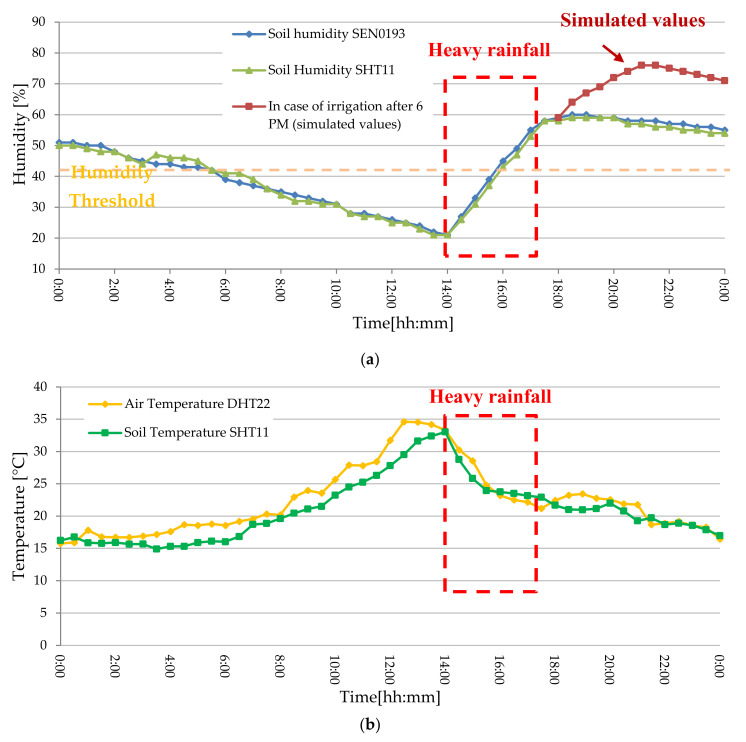
(**a**) Soil humidity temporal trends acquired on land cultivated with tomatoes (on 22/07/2019, Lecce, Italy); (**b**) soil and environmental temperature trends acquired by SHT11 and DHT22 sensors on the same day.

As previously cited, the developed system schedules irrigation and fertigation processes according to the crops need and WSN data, reducing the occurrence of under- or over-watering conditions, thus minimizing any plants stress. In Figure 24a, the temporal trend of soil humidity in the sector 2 is shown; the irrigation was scheduled for 6:00 P.M. (post meridiem), if the soil water content (average value of the data provided by the two sensors) was lower than the set threshold (40%, Figure 24a). However, from 2:00 P.M. to 5:00 P.M. a heavy rainfall occurred, thus the soil humidity exceeded the 40% value, and the scheduled irrigation was canceled, so obtaining water-saving and avoiding tomatoes-plants stress due to overwatering. In fact, in Figure 24a the simulated humidity values, if the irrigation had not been canceled, are reported, calculated taking into account the soil composition (i.e., soil hydraulic conductivity K equals to $7.0\times 10^{-9}\;{\mathrm{ms}}^{-1}$), and the amount of precipitated water using the Darcy’s law [74]. For the same day, in Figure 24b, the temporal trends of soil and environmental temperature are shown; such parameters along with the status of the fertigation system are shown to the farmer through the mobile application, as above described (Figure 19a).

In addition, the developed farm management facility was tested inside a 1700-m^2^ greenhouse where lettuce plants were cultivated; the greenhouse was equipped with suspended sprinkler irrigation implant and a centralized dehumidifier to control the environmental temperature and humidity for optimizing the growth conditions as well as reducing rotting and the spread of diseases and pests. Specifically, the greenhouse was divided into three sectors each equipped with three sensor nodes, one of which configured also as coordinator node; a fertigation unit with a 500-L mixing chamber and a 3-hp pump (model HF-8B) was installed to irrigate three sectors consecutively.

**Figure 25:**
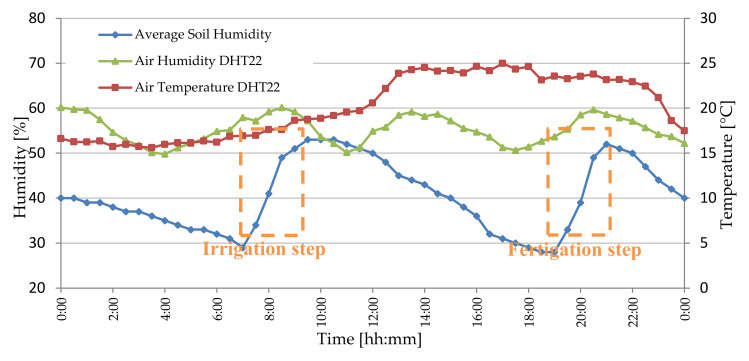
Soil and air humidity and air temperature temporal trends acquired by the wireless sensor network (WSN) nodes installed in a greenhouse, equipped with a centralized dehumidifier implant controlled by the data collected on-field (on 09/09/2019, Lecce, Italy).

The tests were carried out on 9 September 2019; the soil inside the greenhouse is mainly sandy (i.e., with a low TAW—total available water value) and the lettuce plants are next to harvesting phase; therefore, the agronomic database scheduled, for each day, an irrigation in the morning (at 7:00 A.M., Ante Meridiem) and a fertigation process in the evening (at 7:00 P.M.) for a time duration of 30 min (corresponding to 1600 L), if the average soil humidity was lower than 40%. The fertigation consisted of dissolving 1.7 kg soluble N–P–K fertilizer, with 15–5–20 ratios, into the water amount of a normal irrigation. In Figure 25, the temporal trend of soil humidity (blue line) is shown and the effects of the irrigation/fertigation steps are evident. In addition, the detected air temperature and humidity values (red and green lines, respectively) are used to control the dehumidifier implant, so that the greenhouse environmental parameters can be kept inside the set ranges (i.e., 15–25 °C and 50–60%, respectively for temperature and humidity, Figure 25).

## 6. Conclusions

Thanks to the recent availability of advanced hardware and software solutions that allow farmers to control in real time the growth, the harvest and subsequent transformation of cultivated crops, the agricultural companies can increase yields, nutritional quality and safety, from the health point of view, of the cultivated products, reduce production costs and negative impacts on the health and environment, in line with the modern principles of economic, environmental and social sustainability. A management and traceability system, that employs modern technologies, was developed able to support the activities of farms in their productions as well as users of the cloud platform in controlling the cultivation processes directly on-field and to trace the products harvested from the land along the entire production chain. A fertigation system was developed, to manage the frequency and typology of the irrigation processes, controlled by an on-cloud software platform implementing a decision-making algorithm based on the information provided by the weather service and a WSN installed on the field. Specifically, the decisions on timing and fertilizer amount are based on a complete state-of-the-art agronomic database, which represents the smart section of the fertigation system, reproducing, for a given crop typology, the needs in terms of water and fertilizers as a function of the season, growth phase, soil typology, along with the soil and environmental parameters. The information in the agronomic database was determined by collecting and synthesizing the results of previous scientific works and well-known agronomic models (such as the nutrient balance-NB one) and made available to the software platform by means of an on-cloud MySQL database. The integration of the fertigation system with a custom agronomic database represents the main innovation of proposed work. The designed WSN node is energetically autonomous thanks to a solar harvesting system properly sized; also, the sensor node firmware architecture was discussed, as well as the operating modalities for managing data acquisition from sensors and the communication towards the WSN coordinator node. In particular, the power consumption of the WSN nodes was minimized exploiting the built-in low-power modalities of the NodeMCU board and properly configuring the timing of the acquisition and communication phases, along with the status of the connected sensors. Experimental tests were performed for estimating the node power consumption in the different operative phases, obtaining a long lifetime of the used LiPo battery (more than 13 days without any availability of solar energy, an unrealistic extreme case).

In this way, an automated and traced management of the treatments performed on a particular land section is obtained, reducing water and fertilizer waste, as well as minimizing the stress-induced on the plants, thus enhancing the crop yield. A suitable mobile app allows users to fully control all the functionalities of the fertigation system, but also to verify all the treatments performed on a given land section. In addition, the developed fertigation system enables the implementation of specific management strategies, aimed at anticipating or delaying the product collection, actuated by properly tuning the irrigation/fertigation timing, on the basis of the customers’ requests; this scientific innovativeness allows the farmer to achieve economic benefits as well as a reduction of the agri-food waste.

A tracking and tracing system, based on BLE smart tags, was designed capable to monitor the movements of the agri-food products from the cultivation field to the consumers and their storage conditions. To reduce the power consumption of developed BLE tag, commercial microcontroller and sensor breakout boards were modified, resulting in a long-time reusable device, a novelty compared to competitor systems reported in the scientific literature as well as on the market. The chemical/physical quantities detected by the BLE smart tag were chosen in order to provide reliable markers of the freshness status of different product typologies (vegetables, meat, fish, etc.), making it a versatile instrument adaptable to different scenarios. Respect to existing scientific works where only one physical or chemical quantity is monitored to detect the spoiling of the specific stored product, instead, the designed BLE tag is able to simultaneously detect several quantities, so covering a wide range of applications. In addition, the threshold values involved in the HM-10 firmware have to be set as a function of the food typology, amount of the stored product, as well as the typology and size of the used container; in this way, the designed BLE tag can be easily adapted, after a prior characterization, to every container typology. Furthermore, by optimizing the HM-10 firmware, both in iBeacon and acquisition modalities and implementing proper operating strategies, a reduced power consumption of the BLE tag was ensured.

The prototype of the BLE smart tag, based on the CC2541 IC and low-cost sensors, was tested to validate its operation and determine the energy autonomy (up to 5.6 months), value to our knowledge not achieved by other devices present in literature, considering also that our BLE tag allows detecting different chemical/physical quantities, as already highlighted before. The user can remotely access the tracking and storing information by means of a properly developed mobile application synchronized with the on-cloud traceability database. Several tests were carried out to verify the tag functionalities; specifically, we have reported the results related to the BLE tag applied to a closed plastic or polystyrene box with inside oranges or codfish fillets, respectively and monitoring the interest parameters for 30 days (20 days in the refrigerator and 10 days outside) by employing the developed mobile application.

## Figures and Tables

**Table 1:** Absorbed current and time duration values, extracted by the oscilloscope traces, related to the different node operating phases.

Sensor Node Modality	Voltage Drop [mV]	Absorbed Current [mA]	Time Duration [s]
**Deep-sleep**	0.296	0.067	1800
**Searching for coordinator node**	81.424	20.156	3.1
**Acquisition and transmission data to the coordinator node**	160.740	35.125	5.6

**Table 2:** Current absorbed by the BLE smart tag during the different operative phases along with the relative time durations referred to a single operative cycle.

BLE Tag Modality	Absorbed Current [mA]	Time Duration [s]
**iBeacon**	0.237 ^1^	86,400
**Heating of gas sensors**	28.287	600
**Data acquisition and transmission to the Bluetooth gateway**	8.675	1.426

^1^ The current value reported for the iBeacon modality is a mean value over the advertising period (546.25-m-long), with a peak current during the packet transmission equals to 13 mA and a sleep current of 120 µA.
